# The hierarchical assembly of septins revealed by high-speed AFM

**DOI:** 10.1038/s41467-020-18778-x

**Published:** 2020-10-08

**Authors:** Fang Jiao, Kevin S. Cannon, Yi-Chih Lin, Amy S. Gladfelter, Simon Scheuring

**Affiliations:** 1 Department of Anesthesiology, Weill Cornell Medicine, New York, NY 10065 USA; 2 Department of Physiology and Biophysics, Weill Cornell Medicine, New York, NY 10065 USA; 3 Department of Biology, University of North Carolina and Chapel Hill, Chapel Hill, NC 27599 USA; 4 Marine Biological Laboratory, Woods Hole, MA 02543 USA

**Keywords:** Atomic force microscopy, Atomic force microscopy, Nanoscale biophysics, Cytoskeleton, Atomic force microscopy

## Abstract

Septins are GTP-binding proteins involved in diverse cellular processes including division and membrane remodeling. Septins form linear, palindromic heteromeric complexes that can assemble in filaments and higher-order structures. Structural studies revealed various septin architectures, but questions concerning assembly-dynamics and -pathways persist. Here we used high-speed atomic force microscopy (HS-AFM) and kinetic modeling which allowed us to determine that septin filament assembly was a diffusion-driven process, while formation of higher-order structures was complex and involved self-templating. Slightly acidic pH and increased monovalent ion concentrations favor filament-assembly, -alignment and -pairing. Filament-alignment and -pairing further favored diffusion-driven assembly. Pairing is mediated by the septin N-termini face, and may occur symmetrically or staggered, likely important for the formation of higher-order structures of different shapes. Multilayered structures are templated by the morphology of the underlying layers. The septin C-termini face, namely the C-terminal extension of Cdc12, may be involved in membrane binding.

## Abstract (web summary)

Septins are GTP-binding proteins involved in diverse cellular processes including division, polarity maintenance and membrane remodeling. Here authors use high-speed atomic force microscopy to show that assembly of septin filaments is a diffusion-driven process, while septin assembly into higher-order involves septin self-templating

## Introduction

Septins are a family of cytoskeletal GTP-binding proteins, conserved from fungi through humans^1^ that have key roles in cell division, cell polarity, membrane scaffolding and remodeling^2–4^. Due to their diverse and crucial involvement in physiological processes, misregulation of septins has been related to cancers^5^, neurodegenerative diseases^6^, microbial infections^7,8^, and infertility^9^.

All septins share a globular core consisting of a polybasic domain that has been suggested to function in lipid binding, a central GTP-binding domain (G-domain), and a septin unique element^1^. The core is flanked by variable C- and N-terminal extensions, with a notable exception in yeast being Cdc10, which has only a very short C-terminus (alike other mammalian septins of the SEPT3 group)^10–12^. Septins assemble into non-polar filaments through interactions between either adjacent G-domains (G-interface) or through N- and C-terminal helices from the globular core (NC-interfaces)^11^.

The four essential septins in *Saccharomyces cerevisiae* form a linear palindromic hetero-octamer with the following organization: Cdc11-Cdc12-Cdc3-Cdc10-Cdc10-Cdc3-Cdc12-Cdc11^12^. We define this octamer unit as the unitary septin rod, which has a length of ~32 nm, and refer to it as rod in the remainder of the manuscript. These rods further assemble into filaments, filament pairs, and other higher-order structures^12,13^. In vivo, higher-order structures form at sites of cell division^14,15^, and act as scaffolds to recruit and interact with the actomyosin network that drives membrane ingression^16,17^.

Septin function is often associated with filament formation^18^, yet the mechanism of assembly is still unclear. Insights into the septin polymerization process have come from in vitro reconstitution methods using recombinantly expressed fluorescence-tagged septins, planar supported lipid bilayers, and total internal reflection microscopy (TIRF) or FRET-based measurements in solution^19,20^. These microscopy-based data led to a model of diffusion-driven annealing for septin assembly, where septins first associate with the membrane, and through diffusion, collide end-on to form linear polymers^19^. Based on observations including filament length distribution, fragmentation events along the polymer, and the non-helical crystal structure of the human septin complex, it was proposed that septin assembly follows an isodesmic growth process. However, due to the limitations in lateral resolution, it was not possible to follow the earliest stages of septin filament formation from single rods. Thus, the fundamental mechanisms initiating septin polymerization remain poorly understood.

Although septin filaments are found associated with lipid membranes and the cytoskeleton, only the unitary rod is found in the cytosol of fungi and mammals^19,21,22^. How septin rods associate with membranes and the cytoskeleton to promote filament formation is unclear. Up until recently, the septin polybasic domain had been suggested to be responsible for septin-membrane interactions^23^. However, the yeast septin Cdc12 contains a conserved amphipathic helix (AH) within its C-terminal extension (CTE) that binds membranes and is necessary for curvature sensing of septins^24^. Interestingly, the polybasic and AH domains are located on opposite faces of the septin complex, raising questions as to how septins align on the membrane to promote septin-membrane and septin-protein interactions that give rise to the array of higher-order septin structures^23,25,26^.

Understanding the fundamental mechanism of polymerization has been critical for understanding the biophysical properties and functions of the actin and microtubule cytoskeletons and thus the question how septins polymerize is a critical unsolved problem.

Here, we investigate, using high-speed atomic force microscopy (HS-AFM) with molecular resolution, how septins polymerize and assemble into filaments and higher-order structures. We show that septin filament assembly is sensitive to environmental pH and ionic strength. The process is diffusion-driven, further favored by filament-alignment and -pairing. Pairing, mediated by the filament face exposing the N-termini, displays structural variability which is likely important in the formation of higher-order structures of varying shape. The filament face exposing the C-termini, notably the C-terminal of Cdc12, is evidenced to interact with lipids. Finally, septin assembly into higher-order structures involves templating by the underlying septin architecture.

## Results

### HS-AFM allows detection of single septin molecules

High-speed atomic force microscopy (HS-AFM) is a powerful tool for the investigation of biomolecular structures and dynamics such as protein assembly at high spatio-temporal resolution, ~1 nm lateral, ~0.1 nm vertical and ~100 ms temporal resolution, in physiological buffer and at ambient temperature and pressure^27–33^. Here, we took advantage of HS-AFM to visualize the assembly process of recombinantly-expressed septins from the budding yeast, *Saccharomyces cerevisiae*.

**Fig. 1:**
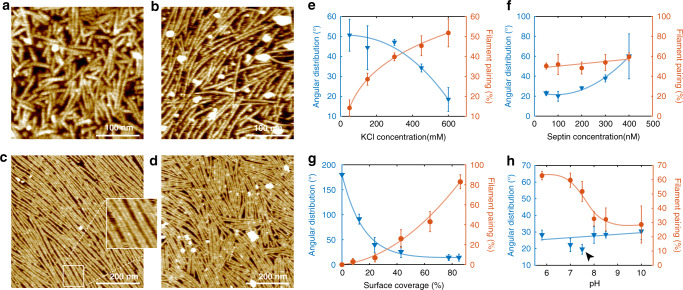
Septin filament assembly, alignment and pairing revealed by HS-AFM. **a** Disordered septin filaments formed using 100 nM septin incubated for 10 min on a hydrophobic epoxy surface in presence of 600 mM KCl. **b** Septin filaments formed using 100 nM septin incubated for 10 min on mica in presence of 150 mM KCl. **c** Septin filaments formed using 100 nM septin incubated for 10 min on mica in presence of 600 mM KCl. Filaments display high packing order and pair formation. **d** Septin filaments formed using 400 nM septin incubated for 10 min on mica in presence of 600 mM KCl. Filaments pair but display limited orientation alignment. **e**–**h** Filaments higher-order analysis, filament alignment (i.e., angular distribution, where a low angular distribution corresponds to a highly ordered packing) and filament pairing as a function of KCl concentration (**e**), septin concentration (**f**), surface coverage (**g**) and pH (**h**). The standard condition of all data collection was 600 mM KCl, 100 nM septin, pH 7.5 and at a surface coverage of ~70%. The solid lines in panels **e**–**h** are spline fits (except for the linear fit of the angular distribution in **h**) to show the general trends of the data within the measurement conditions ranges). Data presented in **e**–**h** are mean ± s.d. from ≥3 different experiments.

We began by sampling different surfaces conducive for HS-AFM to evaluate if and how septins assemble. First, we deposited septin rods on hydrophobic epoxy or silicon surfaces and acquired high-resolution data in which individual septin subunits were clearly resolved along rods and filaments (Fig. 1a; Supplementary Movie 1). These filaments were rather short, with length ~100 nm, thus composed of only ~3 rods. We tested a range of KCl-concentrations (from 150 mM to 600 mM) but did not succeed to visualize filaments of increased length or in higher-order assemblies on such hydrophobic surfaces. In contrast, on mica, which shares some properties of the yeast inner membrane leaflet surface such as hydrophilicity and negative charge, septin rods readily assembled into long filaments with lengths up to several micrometers (Fig. 1b, c), under certain conditions filaments readily paired (Fig. 1c, inset). This suggested that the mica support allowed septin filaments to assemble into higher-order structures. Thus, while future experiments on close-to-native bilayers will allow to decipher the details of the protein-membrane interactions, we decided to use this experimental platform to investigate septin filament formation and supramolecular assembly as a function of environmental factors such as monovalent ion (KCl) concentration, bulk septin concentration and pH (Fig. 1c, d). Note that in all experiments the filament orientation does not coincide with either the fast- (horizontal) or the slow- (vertical) scan axis of the HS-AFM scanning, and thus is not influenced by the imaging mechanism. According to previous studies, septin rods are stable in both high and low salt solutions^12,34,35^; in agreement, we rarely found objects smaller than the septin rod in our in situ HS-AFM frames (Fig. 1). Only in absence of monovalent ions, rod fragments and single septin subunits (<32 nm) could be observed (Supplementary Fig. S1; Supplementary Movies 2, 3).

We first set out to examine septin assembly as a function of varying KCl concentrations from 50 mM to 600 mM (Fig. 1e). Septin filaments assembled well at 50 mM KCl, but they displayed little filament pairing and the angular distribution of the filaments on the surface was wide, *i.e*. the packing order poor (Supplementary Fig. S2a). In contrast, at increasing KCl concentration (up to 600 mM KCl) septin filaments were more and more prone to form pairs and align to each other (Fig. 1c; Supplementary Fig. S2). These findings appear in contrast with a previous negative stain EM report where septins remained heterooligomeric rods when imaged from high-salt buffers in solution (>300 mM) and elongated filaments were visualized from preparations dialyzed against low-salt buffers (<100 mM)^12^. However, this discrepancy can be explained by the fact that we image septin assemblies on the mica surface and not in solution. Our control experiment using negative stain EM corroborate that in high salt concentration no filaments were found in bulk, while they formed at lower salt concentration either in bulk or were able to assemble during the negative staining procedure on the EM grid (Supplementary Fig. S3). In our HS-AFM experiments filaments formed on the surface, and as salt concentrations were raised, electrostatic shielding of repulsive charges on the surface and/or between adjacent septin filaments likely promoted polymerization and pairing. While these conditions are higher ionic strengths than physiological bulk concentrations, we suggest that the relative charge between bulk and the surface is likely what is relevant and thus the high salt favors assembly on the mica, which is more negatively charged than the plasma membrane.

### Diffusion-driven septin filament assembly

We next investigated how changing the concentration of septins influences the organization of higher-order assemblies (Fig. 1f). We found that increasing septin bulk concentration leads to decreased filament alignment, i.e., increased angular distribution (Fig. 1d), while it had no significant effect on filament pairing. This finding is typical for a diffusion-limited elongation process at a solid-liquid interface, where the increased availability of rods in the bulk to form seeds on a surface results in growth of locally aligned filaments without overall long-range order within the boundaries of a diffusion-driven annealing process. Thus, the degree of order within a large-scale higher-order septin assembly can be influenced by the surface deposition rate and molecular diffusivity, which are critical for the ensemble organization of septin structures.

**Fig. 2:**
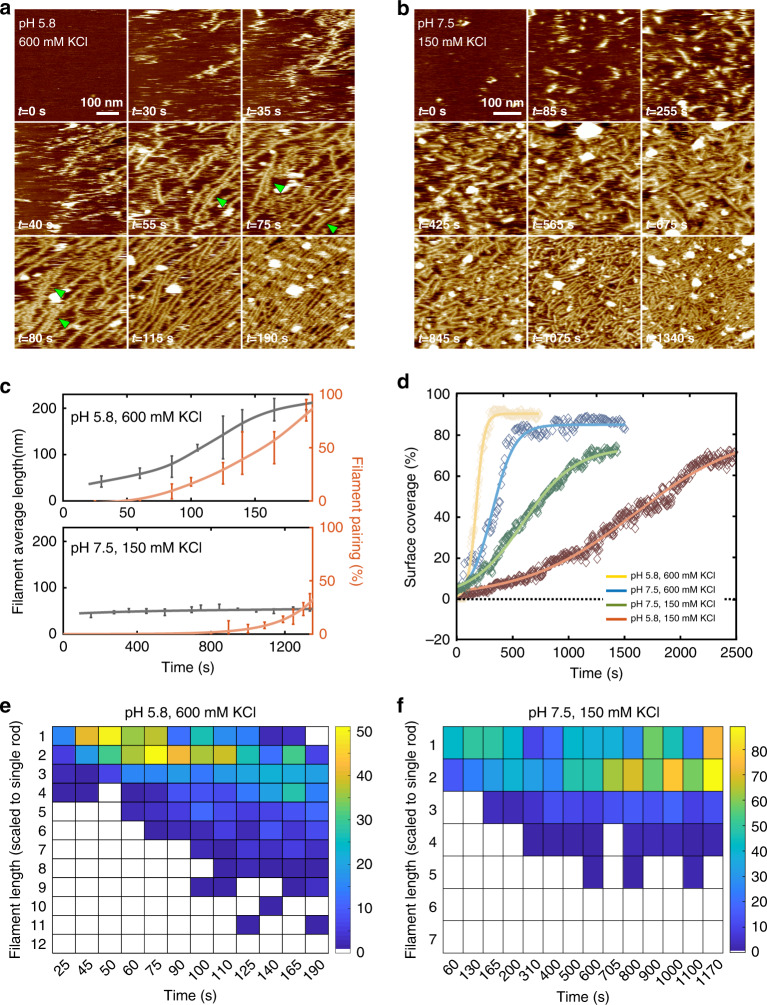
Kinetics of septin assembly imaged by HS-AFM. **a** In situ HS-AFM frames in pH 5.8, 600 mM KCl (top left: imaging conditions; bottom left: image acquisition times) of the septin filaments assembly process. Filament alignment and pairing (green arrows) are established simultaneously. **b** In situ HS-AFM frames in pH 7.5, 150 mM KCl (top left, imaging conditions; bottom left: image acquisition times) showing a much slower assembly process resulting in shorter and less well aligned filaments. **c** Filaments higher-order analysis for (top: **a**) and (bottom: **b**), respectively. Data are presented as mean ± s.d. **d** Surface coverage as a function of time under varying conditions (150 mM or 600 mM KCl in pH 5.8 or pH 7.5 buffer). The solid lines are fitted sigmoidal curves. **e**, **f** Length histograms of septin assemblies as a function of time under conditions **a** shown in **e**, and **b** shown in **f**. Filament lengths were scaled to the rod length, 32 nm. False color bar: Filament count at each time point.

We next assessed the relationship between septin surface coverage, i.e., the encounter probability, and filament alignment and pairing. We hypothesized that pairing of filaments should be enhanced by the abundance of rods and filaments. We performed these experiments at 100 nM septin because higher bulk concentrations reduced long-range filament order. Pairing was limited at initial low surface coverage, where septin rods or short filaments formed at early stages of polymerization deposit in all orientations on the surface. However, with increasing surface coverage, filaments aligned and frequently paired (Fig. 1g). This suggests that long and aligned filaments sample more efficiently and find neighboring filaments for pairing, likely through avidity, where the engagement in many interactions favors further pairing. Additionally, individual septin filaments can serve as a template for the growth of a new filament, thereby creating paired filaments de novo (Fig. 2a, green arrows). Thus, the ability to pair is enhanced at higher septin filaments density through increased encounter frequency, engagement in bonds and/or templated-assembly.

### Septin filament pairing is sensitive to physiological pH

The presence of pairs (Fig. 1c, inset) rather than variable-sized filament bundles suggests that pairing is not simply due to crowding and alignment but is mediated by specific interfaces, as has been suggested by EM^36^. When changing the pH in the HS-AFM fluid chamber, we noticed that septin filament alignment was roughly pH-independent. In contrast, filament pairing was strongly pH-dependent (Fig. 1h). Pairing was favored at slightly acidic pH and disfavored at basic pH, with an inversion point around physiological pH. In addition, the only data point that is a notable outlier in the filament alignment analysis is also at pH 7.5, where best packing homogeneity was found (Fig. 1h, arrow). These results suggest that below pH 7.5 protonation of amino acids on the septins surface promoted filament pairing, thus suggesting that regions involved in pairing are protein moieties rich in Asp, Glu, and His. Moreover, the pH-dependence of filament pairing was not affected by the density of septins on the surface as we observed a lower frequency of filament pairs at higher pH despite high surface coverage (Fig. 1g, h). The fact that the pairing propensity is sensitive around pH 7.5 leads us to hypothesize that minor, physiologically-relevant pH changes might be a means to regulate higher-order septin assembly in cells.

Altogether, the results indicate that septin filament assembly, stability, packing and pairing are modulated by charge and kinetics. Protein surfaces that contain negatively charged amino acids may engage in filament pairing and be charge-neutralized through protonation (at lower pH, Fig. 1h) and/or electrostatic shielding (at high KCl, Fig. 1e). Filament alignment occurs through two-dimensional orientational adjustments, whereas filament pairing and the ultimate long-range order in the assemblies depend on the number of initial molecular deposition sites and the assembly kinetics (Fig. 1f, g).

### Septin assembly kinetics

We next investigated the assembly kinetics in time-lapse HS-AFM experiments. Based on the results of the experiments shown in Fig. 1, we monitored assembly at pH 5.8 or pH 7.5 and in the presence of 150 mM KCl or 600 mM KCl (Fig. 2; Supplementary Fig. S4). As a reminder, lower pH and higher salt concentration favored filament assembly and pairing. The septin concentration was kept constant at 60 nM in all experiments, which is in the same order of magnitude as physiological concentrations^19^.

In elevated ionic strength buffer at slightly acidic pH, filament elongation and pairing occurred basically concomitantly with the rapid surface-binding of septins (Fig. 2a; Supplementary Movie 4). Once filaments were assembled, additional filaments grew in their vicinity and formed pairs (Fig. 2c, top). The entire process was completed within ~3 min and led to the formation of long, well-aligned filaments (Fig. 2a, c). The assembly process critically depended on the surface coverage rate (Fig. 2d, yellow trace) and the subsequent filament growth through surface diffusion (Fig. 2e), as we never observed filaments in solution under similar conditions when investigating the bulk by negative stain EM (Supplementary Fig. S3a).

The length histogram (Fig. 2e) highlights several features in the evolution of septin assembly. First, the most populated septin filament length rapidly shifts from single rod (25–60 s) to short filaments with length ≥100 nm (a detailed filament length analysis of *t* = 190 s is shown in Supplementary Fig. S5). Notably, septin single rods are not found anymore on the surface at the end of the growth experiment (Fig. 2e, right of first row), indicating that new rod adsorption directly advances growth of preexisting filaments. Second, longer filaments, >100 nm, develop mainly after the most populated size was larger than two rods (*t* = ~75–90 s), and their existence distorts the shape of the length distribution with a positive skewness for a long tail, where the longest filaments exceed 300 nm (limited by the scan area for high-resolution HS-AFM movie acquisition). The length-distribution skewness provided evidence that the mechanism of septin surface self-assembly comprised not only diffusion-driven end-on annealing of rods to filaments, but also annealing of filaments to pre-existing filaments^37^. In these conditions, the average growth rate peaks at *t* = ~80 s (Fig. 2d), concomitant with the onset of filaments alignment and pairing (Fig. 2a, c, orange trace). Thus, we propose that well-aligned filaments promote growth of other filaments by reducing the dimensionality of diffusion from two to one in the confined space and through pairing (Fig. 2a, green arrowheads). Finally, the septin filaments on the surface were stable and no fragmentation events were observed after the surface was fully covered. Given the enhanced surface coverage under conditions that promote filament pairing (low pH and/or high salt), we speculate that pairing may influence assembly kinetics by serving as polymerization templates for new septin filaments (Fig. 2a, green arrows).

These analyses were also performed on septin assembly movies in low salt buffer and neutral pH conditions, where septin surface recruitment and assembly was much slower and final filaments were shorter and not well aligned (Fig. 2b, Supplementary Movie 5). Filament average length was nearly constant and remained very short during the entire assembly process and pairing only occurred after ~15 min incubation and only to a minor extent (Fig. 2c, bottom). The low ionic strength buffer critically delays the surface coverage rate (Fig. 2d, green trace), and the length histograms (Fig. 2f) display filament growth rate is slow and only few medium-long filaments ~120 nm (~4 rods) develop in such conditions. In the final state, the observed filaments lie in all orientations and are short, the most populated length is ~60 nm (~2 rods) and isolated septin rods are still abundant. Polymerization is clearly minimized, leading to surface coverage with non-ordered short filaments and rods which in turn likely further confine lateral diffusivity of adsorbents and thus the elongation of septin filaments is limited. Similar phenomena were observed in low ionic strength buffer at slightly acidic pH (Supplementary Fig. S4), supporting the same idea that decreased salt concentrations lead to slower surface coverage rate (Fig. 2d, orange trace) and decreased assembly (Supplementary Fig. S6), associated with diminished filament order. Surface coverage versus time traces displayed a sigmoidal shape (Fig. 2d), indicating a saturation of the available surface for adsorption was reached. Thus, electrostatics influence the rate of adsorption and subsequent order that emerges in a population of filaments, which in turn impacts the length-scale of filaments and the higher-order assembly.

### Simulations support that filament alignment promotes diffusion-driven annealing

**Fig. 3:**
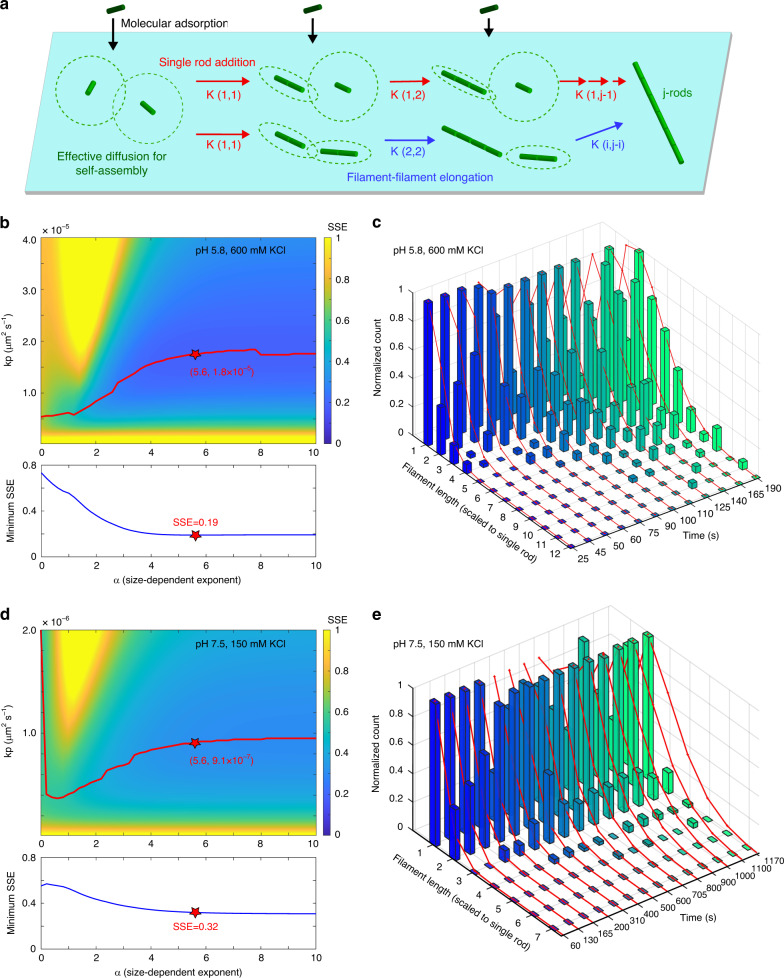
Simulated length histograms based on a diffusion-driven annealing model. **a** Schematic of the diffusion-driven annealing model through single rod addition (red arrows) and filament-filament elongation (blue arrows). The diffusion-driven annealing rate, *K*, depends on the effective diffusion of surface reactants (illustrated by the size of the green dashed circles) for anisotropic self-assembly based on Eq. 1 & 2. **b**, **d** (top) Sum of squared error (SSE) maps between experimental and simulated length histograms, the latter simulated using various *kp* (a diffusion-related constant) and *α* (a size-dependent factor) values, to fit the experimental data at different conditions (600 mM KCl in pH 5.8 buffer (**b**), 150 mM KCl in pH 7.5 buffer (**c**)). The red trace depicts the path of *kp* at different *α* values with minimum SSE. Bottom: The minimum SSE at each *α* value. The red star indicates the best-fit parameters at *α* = 5.6 that offer the global minimum SSE in all conditions. **c**, **e** The comparison between the experimental (bars) and simulated length histograms (red curves), which are calculated by the best-fit parameters at *α* = 5.6.

Leveraging the high spatial and temporal resolution of HS-AFM, we observed septin assembly dynamics from rod surface adsorption to the growth of filaments of various lengths (Fig. 2, Supplementary Fig. S4). The resulting septin length histograms over assembly time (Fig. 2e, f) displayed signatures, i.e., the skewness with a tail corresponding to long filaments, indicative of the balance between molecular adsorption and diffusion-driven annealing (33). Therefore, we investigated if the observed assembly kinetics data could be modeled within the framework of a diffusion-driven annealing model comprising two elementary growth steps, single rod addition and filament-filament elongation (Fig. 3a). The mass balance equation can be expressed as Eq. 1^37^:

1 $$\begin{array}{l}\frac{{\partial f\left( {t,j} \right)}}{{\partial t}} = \frac{\partial }{{\partial t}}\left[ {\frac{a}{{1 + e^{ - b\left( {t - c} \right)}}}} \right] \cdot \delta _{j,1}\\ + 2K\left( {1,j - 1} \right)f\left( {t,1} \right)f\left( {t,j - 1} \right) - 2K\left( {1,j} \right)f\left( {t,1} \right)f\left( {t,j} \right)\\ + \mathop {\sum}\limits_{i = 2}^{j - 2} {K\left( {i,j - i} \right)f\left( {t,i} \right)f\left( {t,j - i} \right)} - \mathop {\sum}\limits_{k = 2}^\infty {K\left( {j,k} \right)f\left( {t,j} \right)f\left( {t,k} \right)}, \end{array}$$

where *f*(*t*, *j*) denotes the surface density (unit: μm^−2^) of septin assemblies of size *j*-rods at time *t*. *K*(*r*, *s*) is a size-dependent, diffusion-driven annealing rate constant for two septin reactants with sizes *r*-rods and *s*-rods, respectively. Equation 1, line 1, describes the adsorption rate of septin rods as depicted by the slope of surface coverage traces (Fig. 2d) fitted by a sigmoidal curve with three parameters, *a*, *b*, and *c* (Supplementary Table S1). Given the known dimensions of the septin rod and the HS-AFM scanning area, we can estimate the rod surface density at any time point as the molecular input for diffusion-driven filament growth steps. Concurrently, septin filament growth is governed by *K*(*r*, *s*) and the surface density of two septin reactants (*r*-rods and *s*-rods) via, single rod addition (Eq. 1, line 2); *r* = 1 and *s* = *j*−1; Fig. 3a, red arrows), and filament-filament elongation (Eq. 1, line 3); *r* = *i*, *s* = *j*−*i*, and *i*, *j* ≥ 2; Fig. 3a, blue arrows). The negative sign of second terms indicates the decrease of the *j*-rod population through the growth steps. The diffusion-driven annealing rate, *K*(*r*, *s*), can be referred to as a generalized sum kernel and expressed as in Eq. 2^37–39^:

2 $$K\left( {r,s} \right) = k_P(r^{ - \alpha } + s^{ - \alpha }),$$

where *k*_*P*_ (unit: μm^2^s^−1^) is a composite factor and related to the diffusivity of septin assemblies for self-assembly, and the changing diffusivity due to alignment of surface reactants, during polymerization. The negative exponent, −α, of the size parameters can be considered as a mobility exponent of septin assemblies of different size. This generalized sum kernel suggests that septin rods and short filaments contribute more to *K*(*r*, *s*) due to their larger effective diffusivity (Fig. 3a, green circle regions), and it simplifies the free fitting parameters to *α* and *k*_P_ in our proposed model.

Using a leapfrog algorithm (see Methods) we simulated the evolution of length histograms under different experimental conditions with various combinations of α and k_P_ values based on Eqs. 1 and 2. We then calculated the sum of square error (SSE) between simulation results and experimental data to determine the goodness of the simulations, and thus determine the best-fit parameters. In the conditions of elevated ionic strength and slightly acidic pH, the SSE map readily indicates a global minimum SSE of 0.19 when *α* = 5.6 and *k*_P_ = 1.8 × 10^−5^ μm^2^ s^−1^ (Fig. 3b, red star). With the best-fit parameters, simulated length histograms (Fig. 3c, red lines) describe well the growth of the most populated septin filament length, and reproduce the positive skewness with a long tail in experimental length histograms (Fig. 3c, colored bars, after 90 s). Herein, the negative size exponent terms in K(r, s) in Eq. 2 plays a critical role to modulate the skewness of the length distributions and enhances the growth of leading length away from the most populated length via filament-filament elongation (Eq. 1, line 3).

At low salt and neutral pH, the SSE map also shows a semi-global minimum SSE of 0.32 when *α* = 5.6 and *k*_P_ = 9.1 × 10^−7^ μm^2^ s^−1^ (Fig. 3d, red star). The relatively large SSE is attributed to the differences between simulation and data where the first predicts the growth of longer filaments after 600 s that are missing in the experimental data (Fig. 3e, color bars). This disparity can be explained by the fact of that our model only considers an ideal case without the impacts of crowding of rods and short filaments of varying orientation in the diffusion-driven annealing rate. Thus, the k_P_ in the low pH/high salt conditions is ~20 times larger than in neutral pH/low salt, which indicates that the good alignment of surface reactants in the first case promotes the polymerization process. We performed the same numerical simulations for the low pH/low salt and neutral pH/high salt conditions (Supplementary Fig. S6), which exhibited different degrees of filament alignment disorder (Supplementary Fig. S4), and received k_P_ values that are proportional to the filament alignment in these conditions and are between the values obtained for the low pH/high salt and neutral pH/low salt conditions (Supplementary Table S2). Therefore, we concluded that the filament alignment order, effectively reducing the diffusive dimension in the diffusion-driven annealing processes, has a strong influence on septin polymerization.

### Evidence for interaction of the Cdc12 C-terminal domain with lipids

**Fig. 4:**
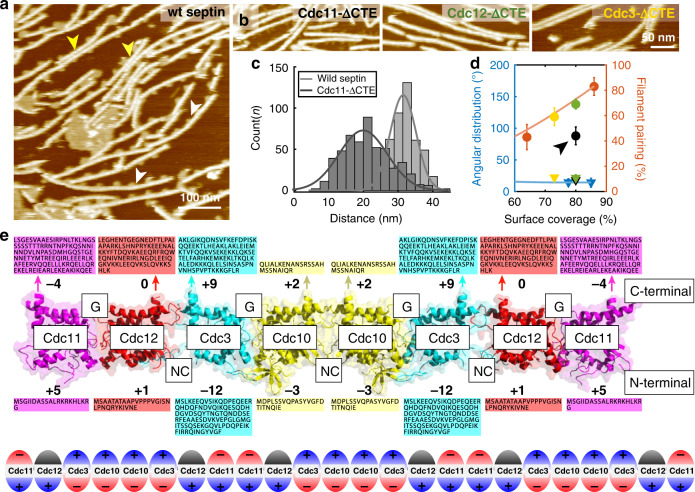
Septin filaments bind lipid with the C-terminal face and pair on the N-terminal face. **a** Wild-type septin filaments periodically recruit small lipid clusters on one side of individual septin filaments (white arrowhead), and on both outer faces of paired filaments (yellow arrowheads). Occasionally, these lipid clusters fused on the surface (center of the image). **b** Septin filaments with C-terminal deletion mutants Cdc11-ΔCTE, Cdc12-ΔCTE or Cdc3-ΔCTE septins: Only filaments containing Cdc11-ΔCTE recruit lipid clusters, but display slightly diminished pairing. **c** Distance distribution between lipid clusters recruited by wild-type septins (~32 nm), and Cdc11-ΔCTE (~20 nm). **d** Filament alignment (triangles) and pairing (circles) containing Cdc11-ΔCTE (black), Cdc12-ΔCTE (green) and Cdc3-ΔCTE (yellow), compared to wild-type filaments. Data are presented as mean ± s.d. from ≥ 3 different experiments. **e** Structural model of the yeast septin hetero-octamer rod generated by aligning of the crystal structure of Cdc11 (PDB: 5AR1), and models of Cdc12, Cdc3, and Cdc10 to the crystal structures of in the human hetero-hexamer rod septin-9 (PDB ID: 5CYP), septin-7 (PDB ID: 2QAG), and septin-3 (PDB ID: 4Z54). Boxes display N- and C-terminal sequences, respective (arrows indicate where C-termini protrude from *α*6 of the respective subunits). The bold numbers indicate the net charges of the surface exposed structurally unresolved termini. Bottom: Schematic of the charge distribution along a septin filament.

When septin filament assembly was imaged at high resolution in high KCl concentrations, periodic dots could be observed at the filament sides (Fig. 4a, white arrowheads). These clusters always occurred on the same side of a given filament and located on the outside of both filaments in paired filaments (Fig. 4a, yellow arrowheads). To our surprise, we also found small lipid clusters in the negative stain EM of the same septin sample (Supplementary Fig. S7), thus we concluded that lipids, its natural binding partner, were co-purified with septins^40–42^. The fact that the lipid clusters located on the outer faces of paired filaments suggested that membrane binding sites and filament pairing sites were mediated by different protein moieties on opposing faces of the septin filaments. To evaluate what part of the septins interacted with lipids, we analyzed the periodicity of the initial lipid clusters, and septin filaments composed of deletion mutants of the C-terminal extensions of Cdc11, Cdc12 or Cdc3 (Cdc11-ΔCTE, Cdc12-ΔCTE, Cdc3-ΔCTE). Similar to wild-type rods (Fig. 4a), only Cdc11-ΔCTE filaments exhibited the clusters, while filaments with Cdc12-ΔCTE or Cdc3-ΔCTE showed bare filaments (Fig. 4b). The periodicity of lipid clusters on wild-type septin filaments was ~32 nm (i.e., the length of the rod), while filaments of Cdc11-ΔCTE revealed an average of ~20 nm between clusters with a somewhat wider distribution (Fig. 4c). We cannot interpret this difference with certainty, but note that a periodicity of ~20 nm in the Cdc11-ΔCTE filaments is consistent with the distance between Cdc12s and/or Cdc3s (both having inter-subunit distances of 16 nm and 24 nm in the palindromic filaments). A recent work identified an amphipathic helix in the CTE of Cdc12^24^, consistent with lipid binding capacity. The lipid clusters appear very close to the filaments in the HS-AFM images, while the amphipathic helix in the CTE could possibly be distant. This is explained with the AFM-tip convolution that gives the filaments a wider appearance and/or with the flexibility of the CTEs and the lipid clusters.

### Cdc12 and Cdc3 C-terminal extensions are not essential for pairing

We next evaluated the filament formation and pairing of the various CTE mutants. The pairing and filament alignment abilities of Cdc12-ΔCTE and Cdc3-ΔCTE mutants were almost identical to wild-type (Fig. 4d, green and yellow), indicating that the C-terminal coiled-coil domains of Cdc12 and Cdc3 did not contribute to filament pairing on mica surfaces. While the Cdc11-ΔCTE mutant filaments fully covered surfaces after extended incubation times with angular alignment similar to the wild-type (Supplementary Movies 6, 7), their pairing ability was slightly impaired compared to wild-type (Fig. 4d, arrowhead), consistent with a previous report^36^. Also, Cdc11-ΔCTE mutant filaments were more fragile and often dissociated during HS-AFM scanning, which resulted in shorter filaments on average (Supplementary Movies 6, 7). Altogether, these results indicate that the C-terminal coiled-coil domain of Cdc11 is implicated in filament pairing and stabilizes the association of the rods along the filaments. Previous work has shown the CTE of Cdc11 was capable of homotypic interactions^43^, in agreement with the diminished pairing ability of the Cdc11-ΔCTE mutant. Moreover, given the predicted flexibility of septin CTEs, we speculate that filament pairing could be stabilized through Cdc11-CTE homomeric interactions that lie perpendicular to the membrane plane. Thus, in the conditions of these experiments, the CTEs of Cdc12, and potentially Cdc3, can associate with lipids but are not required for filament pairing, while the CTE of Cdc11 contributes to the stability of filaments and their ability to form pairs.

### Septin filaments have a patterned charge distribution

The polarity of lipid association and pairing to opposite faces of the septin filament, along with the conclusions provided by the condition-dependent assembly data shown in Fig. 1, prompted us to build a structural model of the yeast septin rod, including the charge properties of the terminal extensions that are not resolved in the X-ray structures (Fig. 4e). First, we aligned the structures of yeast Cdc3, Cdc10, and Cd12 with the human septins septin-7, -3, and -9 (PDBs: 2QAG, 4Z54, 5CYP), of which the structures were solved in the context of the hexameric human rod (Supplementary Fig. S8a). Adding to this hexameric rod model the yeast Cdc11 (PDB: 5AR1)^43^ through structural propagation of the G-interface as in between Cdc10 and Cdc3, we built a full yeast septin rod structural model and investigated the surface exposed charges and found that the modeled yeast septin hetero-octameric rod showed nearly neutral surface charges on all faces (Supplementary Fig. S8b). However, all these structures lack the N- and C-terminal extensions. Thus, we analyzed the N- and C-terminal sequences extending from the structural model and calculated the net charges of these unresolved structural elements (Fig. 4e). We found that Cdc3 and Cdc11 had very interesting charge distributions of the N- and C-termini with −12/+9 for Cdc3 and +5/−4 for Cdc11 (and to a lesser extent Cdc10 with −3/+2). Importantly, if these charge distributions along the rod are considered in the context of a long palindromic filament, an alternating pattern of inverted charge distributions is to be expected (Fig. 4e, bottom). In such a filament context, positive charges dominate around Cdc3-Cdc10-Cdc10-Cdc3 intercalated by negative charges around Cdc11-Cdc11 on the C-terminal side, and inverted charges on the N-terminal face.

From the experiments varying environmental pH and ion concentration shown in Fig. 1, we know that regions involved in filament pairing are protein moieties rich in negatively charged amino acids, which are found in the N-termini of Cdc10 and Cdc3. On the other hand, filaments comprising C-terminal deletions of Cdc12 or Cdc3 could not recruit lipid clusters. These data suggest the filament pairing face is the N-terminal and the membrane binding is the C-terminal face. Note, the overall net charge of the C-terminal face is positive and thus favorable to bind to negatively charged lipids of the inner membrane leaflet. This arrangement places the strongly negatively charged N-termini of the Cdc3-Cdc10-Cdc10-Cdc3 stretch on the other face, ideally to engage into filament-filament interactions. The flexibility of the CTE of Cdc11 may contribute to inter-filaments contacts, as its absence weakens filament paring ability (note, it is also slightly negatively charged).

### Variability of filament pairing interactions

**Fig. 5:**
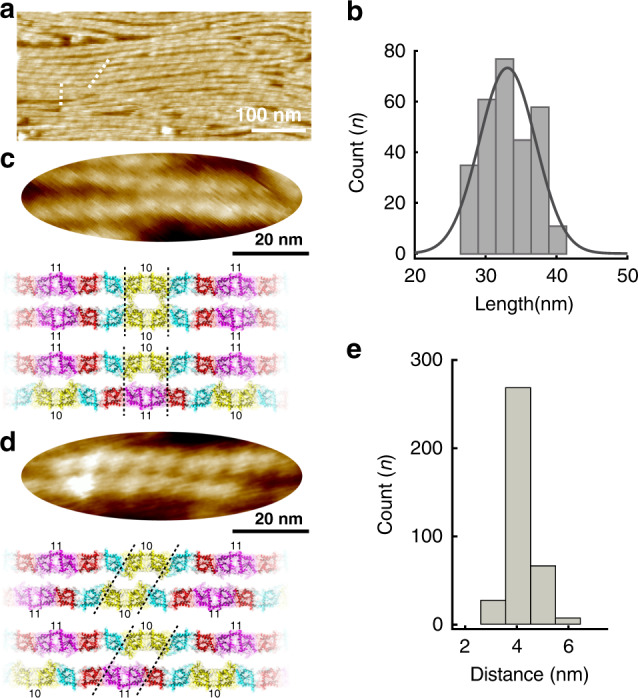
Variability of filament pairing. **a** Septin filament pairing exhibit subunit interaction match and mismatch in the same field of investigation. **b** Periodicity length distribution of interacting elements peaking at ~32 nm, i.e., the length of the hetero-octameric septin rod. High-resolution images of **c** matched and **d** mismatched filament pairs. Bottom in **c** and **d**: Structural models of matched and mismatched rod interactions. The HS-AFM resolution does not allow to distinguish the precise nature of the septin subunit, cases where Cdc10 (label 10) and/or Cdc11 (label 11) face each other are illustrated. **e** Histogram analysis of the top-to-top distance of the filaments in filament-pairs peaking at ~4 nm.

Given that filament pairing occurred early and should therefore be an important step in the formation of higher-order septin structures, we next investigated molecular-level details of the inter-filament interaction (Fig. 5a). As expected, we found a uniform length periodicity of ~32 nm along all filaments, representative of the length of the hetero-octameric rod (Fig. 5b). In agreement with previous EM studies^12^, we found filament pairs where the subunit periodicity of the two interacting filaments matched (Fig. 5a, vertical dashed line; Fig. 5c), while other filaments paired with a phase shift, i.e., subunits of interacting filaments were mismatched (Fig. 5a, tilted dashed line, Fig. 5d). The mismatch distance was about 4 nm, corresponding to the length of one septin subunit, i.e., half of the distance between NC-interfaces. Based on these observations, we propose alternative models where septin filament pairing is mediated by N-terminal domains precisely facing each other or in a configuration where they intercalate at the NC-interface locations. The HS-AFM resolution is however only molecular, and we cannot distinguish which subunits face each other. Indeed, given that Cdc10 and Cdc11 have opposite charges on the N-terminal faces, it is appealing to suggest that these subunits pair in the matched filament pairs (Fig. 5c, d, molecular models). While isolated filaments may appear enlarged due to AFM-tip convolution, the precise interfilament distance can be assessed when measuring the top-to-top distance of closely associated filament pairs. These distances were always <10 nm, and thus there was little (if any) space between paired filaments (Fig. 5e). This is different from previous EM observations where neighboring filaments exhibited a railroad track-like appearance with a 15- to 25-nm gap^12^. The variability of filament pairing interactions is likely of importance in the formation of higher-order structures, especially those that are bent. In any bent higher-order structure the filament positioned in an outermore location (with larger radius of curvature) will have a longer path than a filament located closer to the center of a curved structure. Thus, pairing variability may be a prerequisite for septins to accommodate to curved architectures.

### Template-controlled 3D-growth of septin filaments

**Fig. 6:**
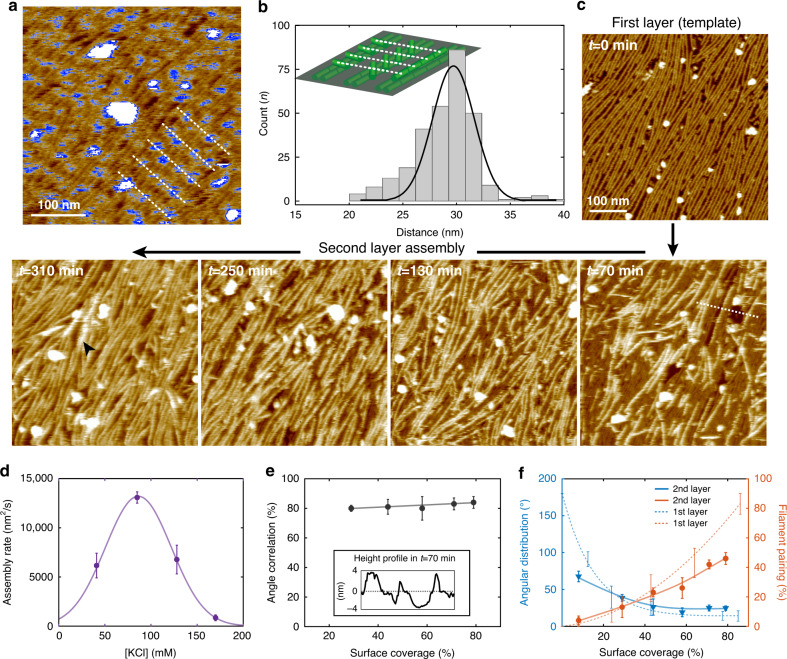
Multilayered septin filament assembly observed by HS-AFM. **a** Contrast adjusted HS-AFM frame of septin filaments on which second layer septin molecules start to adhere on the first layer filaments (false-color scale is adapted to enhance the strongly protruding features in blue; the dashed white the periodicity of the features, roughly perpendicular to the aligned first layer filaments). **b** Distance distribution of recruited second layer septins. Peaking at ~30 nm periodicity, matching the septin rod length and thus suggestive of an interaction with Cdc11 or Cdc10 (Inset: cartoon of second layer septin rods on the first layer filaments). **c** HS-AFM image sequence of a second-layer septin filaments assembly on top of a first layer by injection of fresh septin solution (in low salt buffer: 85 mM KCl). Following second-layer formation initiation of filament assembly for the formation of a third layer was observed (arrowhead in *t* = 310 min). **d** Septin multilayer assemblies can only be established within a rather narrow window of KCl concentration (50 mM to 150 mM KCl). Data are represented as mean ± s.d from ≥3 different experiments. **e** Second layer assembly is guided by the first layer serving as a template: ~80% of the second layer filaments align with the sub-layer filaments, independent of the second-layer filament surface coverage. Inset: Height section profile (along the dashed line in image *t* = 70 min) showing the two layers of ~4 nm in height, where the surface of the first layer is set to 0 nm. Data are represented as mean ± s.d from ≥3 different imaging areas from 1 representative time-lapse experiment. **f** Both packing orientation and filament pairing are strongly influenced by the first septin layer serving as template. Data are presented as mean ± s.d. from ≥3 different imaging areas from 1 representative time-lapse experiment.

Once a surface was fully covered with aligned and paired septin filaments, we detected additional topographic features on top of the layer of filaments (Fig. 6a). These blurry surface features had a periodicity of ~30 nm (Fig. 6b), coinciding well with the length of single septin rods, evidence that either Cdc11 or Cdc10 was involved in the recruitment of an additional protein layer (as a reminder, the repetitive palindromic rod sequence is Cdc11-Cdc12-Cdc3-Cdc10-Cdc10-Cdc3-Cdc12-Cdc11). We interpret these protrusions associated to the base septin filament layer as initial septins that may serve to template a second septin layer.

Thus, we next investigated how septins assembled into 3D-structures using HS-AFM experiments of long duration (Fig. 6c). First, based on our experiments as displayed in Figs. 1 and 2, we let a single layer of highly orientated filaments form as a template (Fig. 6c, *t* = 0 min). Then, fresh septin in low salt buffer was added to the template layer to allow assembly of a second layer. We observed that septin filaments started to assemble on top of the first layer (Fig. 6c, *t* = 70 min), until full surface coverage was reached again (Fig. 6c, *t* = 310 min). After establishment of the second layer, filaments initiating a third layer were observed (Fig. 6c, *t* = 310 min, arrowhead). We found that, in contrast to the formation of the first layer that formed on mica under a variety of conditions but most efficiently at high KCl (600 mM), only a narrow window of low salt concentration (<200 mM) permitted the formation of multilayered hierarchical structures (Fig. 6d). The assembly rate of additional layers was fastest in buffer containing ~85 mM KCl. In general, the assembly rate of the second layer was always lower than that of the first layer. We interpret these findings in the following way: The conditions for the second layer growth is based on native-like protein-protein interactions and performs best under physiological conditions, while the assembly on the mica (that also works under such physiological ion concentrations) is faster under higher ionic strength due to the high surface charge of the mica surface. The templating relationship between first- and second-layer filaments is quantitatively assessed with an ~80% angular correlation (i.e., 80% of filaments in second layer align with the bottom layer), independent of the surface coverage of the second layer (Fig. 6e). Interestingly, we found that the second layer has better angular alignment at low surface coverage than the template layer had during formation (Fig. 6f, left), evidence that the first layer readily serves as an alignment template for a more dispersed second layer of filaments. Upon full second layer coverage, the packing orientation is relatively homogeneous, but not as tight as that of the first septin layer (Fig. 6f, right), and filament pairing is much less pronounced in the second compared to the first layer, likely a signature of establishing vertical interactions at the price of lateral interactions (Fig. 6f, right).

These experiments suggest that the assembly of multilayered architectures is a complex process that is strongly influenced by lateral and vertical interactions between septin layers. While the first layer aligns and pairs almost perfectly, the higher-order assembly is guided by the underlying layer but is overall less ordered, likely leading to a more and more complex and possibly disordered network as more layers are added. The decrease of filament pairing in the second layer indicates that the filament-filament interaction surface between layers and between filaments within one layer are likely the same, thus already occupied by second-layer filaments with their partners underneath not allowing lateral pairing. This concept raises the question, which we cannot answer, whether first-layer filaments remain paired upon growth in 3D.

## Discussion

In this work, we employed high-speed atomic force microscopy (HS-AFM) to investigate the assembly process and structure of septins from single subunits to higher-order 3D-structures. HS-AFM has formerly proven powerful to study assembly, structure and dynamics of membrane associated proteins^27,28,44,45^. Our experiments provide insights into the role of pH and ions in modulating assembly, the mechanism of polymerization, first level higher-order structure formation through filament pairing, and growth into 3D multi-layer architectures. We also provide evidence about the faces of the filaments and how they are involved in lipid interactions and protein-protein interactions. The temporal and spatial resolution of HS-AFM has revealed never before detected features of septin assembly from the scale of the single molecule to layers of filaments in higher-order assemblies.

In the reconstituted setting, electrostatic interactions played a significant role in filament alignment and pairing, where increased shielding of surface charges through the higher abundance of monovalent ions favored aligned packing and pairing. The average length of filaments increased with salt concentration and long-range ordering of assemblies. We suspect that in cells this shielding occurs due to the large network of interacting proteins that are known to associate with septins, some of which are known to impact the integrity of the higher-order assembly. Alternatively, one could speculate that gated transmembrane channels may locally modulate ion conditions at sites where septins are active in certain cell types and conditions. What is clear is that the degree of short-range order in the form of pairing and long-range order amongst associated filaments can be tuned electrostatically.

Another striking control of higher-order assembly can be seen in the pH sensitivity of filament pairing. The sensitivity has an inversion point around physiological pH, which suggests that regions involved in filament pairing are protein moieties rich in Asp, Glu or His. A structural model further indicates that the N-terminal extensions, especially in Cdc3, contain numerous of Asp and Glu, indicative that the face of the septin rod comprising the N-termini engages into filament-filament interactions. Interestingly, the C-terminal extensions of Cdc11 also contains Asp and Glu residues, consistent with our data that show reduced pairing and filament stability in Cdc11-ΔCTE septin complexes. Together these data point to a mechanism by which cells may be able to control septin assemblies using small changes in pH. We hypothesize that pH changes in addition to phosphorylation could potentially play roles in the rapid rearrangements of septins at cytokinesis and could contribute to asymmetry of septin ring disassembly that can sometimes be seen as there is a mother-daughter asymmetry in pH in budding yeast^46,47^.

Understanding the growth mechanism of polymer formation has been central to the study of actin and microtubule cytoskeletons as well as bacterial cytoskeletal polymers such as FtsZ. To this point, there has been very little known about the dynamics of assembly mechanisms of septins aside from what has been gleaned from TIRF-based assays (19), which have limitations due to the resolution of the light microscope. Our previous work suggested an isodesmic assembly process where low protein concentrations were sufficient for polymerization^19^. Here we see evidence for a diffusion-driven annealing process in the fast self-assembly of septin filaments depending on the order of filament alignment. A subtle interplay between the molecular adsorption rate and the diffusion-driven annealing process has a great impact on long-range ordering, and could be a key for controlling size and patterns within higher-order septin assemblies. We suspect that molecular crowding in a real cell membrane setting could fine-tune the dynamics of septin polymerization, through the interaction with other proteins and/or changing the environmental electrostatics and pH, and post-translational modifications that modulate surface charge.

In many systems, septins are closely associated with and thought to modulate lipids including the plasma membrane, the ER, membranes of intracellular bacteria, and lipid droplets^41^. Notably, we saw what appear to be droplets of lipid associated with septin rods. Remarkably, the lipid clusters displayed periodicity of rod-length and were recruited and remained on the same face of a given filament, the outside of both filaments when they were paired. This strongly suggests that at least one region for membrane-binding and filament-filament interaction are on the two opposite faces of septin filaments.

In the conditions of our experiments, we show that filament pairing is guided by the N-terminal face. Furthermore, we only see tightly paired filaments, while by negative stain EM both tightly and loosely paired filaments were observed. Several studies have suggested that the CTEs in several septins participate in both homo-/heterotypic protein interactions, thereby providing a mechanism to regulate higher-order septin structures, including filament pairing^43,48,49^. Tight filament pairs were shown to be independent of CTEs. However, loosely paired filaments have been suggested to be dependent on CTE-CTE interactions^12,50^. Subsequent studies have also shown that the septin interacting protein, Bni5, interacts with the CTE of Cdc11 generating structures reminiscent of loosely paired filaments^49^. In our experiments, analysis of various C-terminal deletion mutations identified membrane-binding to the C-terminal extensions of Cdc3 and Cdc12, while filament-filament interactions are mediated primarily by the N-terminal extensions. A possible explanation for the CTE-dependent formation of loosely paired filaments of Bertin et al.^12^ may be that by virtue of flexible linkers between the globular domain and the predicted coil of Cdc11, this extension may be reached by other septins for loose interactions.

HS-AFM also revealed key features about layered, higher-order assembly. In particular, high-resolution images revealed that filament pairing could occur with subunits facing each other or with subunits offset by one protein, thus placing N-termini of one filament into the groove of the NC-interface of the other filament. Variability of filament interface contacts are likely important for the formation of curved septin assemblies such as gauzes, and rings^26,51^. Multilayered septin filaments can only be established in physiological conditions (<200 mM KCl). Interactions between layers dominate the packing orientation of multilayer filaments, thus lower layers serve as templates for growth into the third dimension. The observation of alignment and pairing within layers and templating between layers may be at the origin of the formation of thick bundles. In cells, the cytoplasmic septin concentration was measured to be 100–200 nM^19^ and the KCl concentration is 100–300 mM, thus the observed mechanism may well be representative of the formation of higher-order structures in cells.

The application of HS-AFM to the septin cytoskeleton has enabled the first dynamic analysis of septin assembly from single subunits to higher-order structures, the morphologies of the hierarchical assemblies and the environmental factors that influence assembly. The data provide important insights at the many-molecules level too large for high-resolution structural techniques and too small for optical approaches typically used for in vivo investigations.

## Methods

### Septin expression and purification

Plasmids encoding either 6-His-TEV site-Cdc12/Cdc10 or Cdc11/Cdc3 (Supplementary Table S3)^19^, we co-transformed into BL21 (DE3) *Escherichia coli* cells and selected for using chloramphenicol and ampicillin. Positive cultures were grown overnight in Luria-Bertani broth, chloramphenicol, and ampicillin. Cultures were then inoculated into one liter of Terrific Broth with chloramphenicol and ampicillin and grown to an O.D._600nm_ between 0.6–0.8. Cells were induced with 1 mM of isopropyl- β-D-1-thiogalactopyranoside for 24 h at 22 degrees Celsius. Cells were pelleted at 13,689 xg relative centrifugal force for 15 min. The pellet was re-suspended in lysis buffer (1 M KCl, 50 mM Hepes pH 7.4, 20 mM Imidazole, 1 mM MgCl_2_, 10% glycerol, 1% Tween-20, 1x protease inhibitor tablet, and 1 mg/ml lysozyme) on ice through vortexing every 5 min for 30 min. The lysate was sonicated twice on ice for 10 s with 2 min between sonication steps. The lysate was clarified through centrifugation in an SS-34 rotor at 47,807 *g* for 30 min. The supernatant was harvested, passed through a 0.44 µm filter, and incubated with 2 mL of equilibrated HisPur cobalt resin at 4 degrees Celsius for 1 h. The resin-lysate slurry was added to a gravity flow column. The resin was washed 4 times in 5 column volumes in wash buffer (1 M KCl, 50 mM Hepes pH 7.4, and 20 mM Imidazole). Septin complexes were eluted using elution buffer (300 mM KCl, 50 mM Hepes pH 7.4, and 500 mM Imidazole) and subject to two-step dialysis for 24 h into septin storage buffer (300 mM KCl, 50 mM Hepes, 1 mM β-mercaptoethanol) using a 100 K molecular weight cut off dialysis cassette. 60 µg of Tobacco Etch Virus protease was added to the cassette cleave the poly-histidine tag on Cdc12. After 24 h, purified septin complexes were run over a second HisPur cobalt resin and the flow through was collected. Protein purity and concentration were assessed using SDS-PAGE and Bradford assay, respectively.

### Sample preparation

The purified protein was diluted to 100 nM septin with buffer containing 25 mM HEPES-NaOH, pH7.5, 600 mM (or 150 mM, and other concentrations of) KCl and 0.5 mM BME. Then 3 μl septin solution was immediately deposited on a 1.5 mm-diameter freshly cleaved mica (clean epoxy or silicon) and incubating for 5–50 min. Sample was gently rinsed with incubating buffer and imaging buffer. In situ assembly experiments were performed through subsequent addition of septin into the HS-AFM fluid cell during operation, to reach 60 nM final concentration. Lipid clusters were co-purified with septin, their observation and subsequent bilayer formation was performed in buffer containing 25 mM HEPES-NaOH, pH7.5, 750 mM KCl, 0.5 mM BME. To image filament pairing details with high contrast, setpin filaments were assembled in buffer containing 25 mM HEPES-NaOH, pH 7.5, 600 mM KCl, 0.5 mM BME, and imaged in high salt buffer conditions 750 mM KCl.

### High-speed atomic force microscopy (HS-AFM)

All images in this study were taken using HS-AFM (SS-NEX; RIBM, Japan) operated in amplitude modulation mode using optimized scan and feedback parameters. The HS-AFM was steered using operation packages in IgorPro (WaveMetrics, USA). Ultra-short (8-μm) cantilevers (USC, NanoWorld, Switzerland) with nominal spring constant of 0.15 N/m, resonance frequency of ~0.6 MHz, and a quality factor of ∼1.5 in imaging buffer (25 mM HEPES-NaOH, pH7.5, 150 mM KCl and 0.5 mM BME). Single septin subunit dissociation and association experiments were performed in 25 mM HEPES-NaOH, pH 7.5, 2 mM CaCl_2_, 0.5 mM BME.

### Negative-stain electron microscopy

Septin samples were prepared by pipetting ~5 µl of complex (following 10 min incubation of 100 nM septin in 25 mM HEPES-NaOH, pH 7.5, 600 mM (or 150 mM) KCl and 0.5 mM BME) onto carbon-coated glow-discharged electron microscopy grids. After 7 min grids were rinsed and negative staining was performed through addition of 2% phosphotungstic acid which was removed with a filter paper after 1 min. TEM was conducted on a 100-kV JEOL 1400 TEM microscope.

### Data analysis

HS-AFM images and movies were drift corrected using a dedicated plugin in ImageJ^52^. Filaments angle distribution data (alignment) was extracted using the OrientationJ plugin in ImageJ, written by Daniel Sage and available at http://bigwww.epfl.ch/demo/orientation/. Surface coverage of in situ experiments was determined by dividing the area covered by filaments with the total area (following thresholding between the mica surface and the thickness of a filament layer). In Figs. 1, 4 and 6, the angular distribution values are the average of the full width at half height (FWHH) of Gaussian fits of the filament orientation vector distributions of 3 images under each condition. The error bars of these values are ±1 standard deviation (SD) of the mean of the values from each image. In Figs. 1, 2, 4 and 6, the filament pairing values are the percentage of total filament length that is paired in each image from 3 images under each condition. The error bars of these values are ±1 standard deviation (SD) of the mean of these percentages from each image. The filament length was manually measured in ImageJ with edge enhancement.

### Numerical simulation

The evolution of the septin filaments was calculated in MATLAB using a leapfrog algorithm based on the recurrence relation (Eq. 3) with Δ*t* = 1 s and *j* = 1–12^37^.

3 $$f\left( {t + {\Delta}t,j} \right) = f\left( {t,j} \right) + \frac{{{\Delta}f\left( {t,j} \right)}}{{{\Delta}t}} \times {\Delta}t$$

Here, we estimate the different septin adsorption rates from the surface coverage curve (Supplementary Table S1) to simulate the evolution of length histograms under varying experimental condition. At the end of the simulation, the population of *j*-mers at various times was normalized to 1, and then compared with the normalized experimental length histograms to calculate the sum of square error (SSE). The reported SSE was scaled to the number of experimental length histograms to eliminate the bias from different sampling size.

### Statistics and reproducibility

All the data showed in Figs. 1a-d; 2a, b; 4a, b; 5a, c, d; 6a, c; Supplementary Figs. 1a–c; 2a–e; 3a, b 4a, b; 5b; 7a. are repeated at least 2 times with similar results.

### Reporting summary

Further information on research design is available in the Nature Research Reporting Summary linked to this article.

## Supplementary information

Supplementary Information

Supplementary Movie 1

Supplementary Movie 2

Supplementary Movie 3

Supplementary Movie 4

Supplementary Movie 5

Supplementary Movie 6

Supplementary Movie 7

Reporting Summary

## Data Availability

Data supporting the findings of this manuscript are available from the corresponding author upon reasonable request. A reporting summary for this Article is available as a Supplementary Information file. Source data are provided with this paper.

## Source data

Source Data
