# Supplementary material for: The hierarchical assembly of septins revealed by high-speed AFM
Source: Nat Commun. 2020 Oct 8;11:5062. doi: 10.1038/s41467-020-18778-x (PMC7545167; doi:10.1038/s41467-020-18778-x)
Supplement: Supplementary file 1 — Supplementary Information [file 41467_2020_18778_MOESM1_ESM.pdf]

The hierarchical assembly of septins revealed by high-speed AFM

Fang Jiao[1,2], Kevin S Cannon[3], Yi-Chih Lin[1,2], Amy S Gladfelter[3,4], and Simon Scheuring[1,2,*]

1. Department of Anesthesiology, Weill Cornell Medicine, New York, NY 10065
2. Department of Physiology and Biophysics, Weill Cornell Medicine, New York, NY 10065
3. Department of Biology, University of North Carolina and Chapel Hill, Chapel Hill, NC 27599
4. Marine Biological Laboratory, Woods Hole, MA 02543

* Correspondence to: sis2019@med.cornell.edu

Supplementary Figures and Information

Supplementary Figure 1)

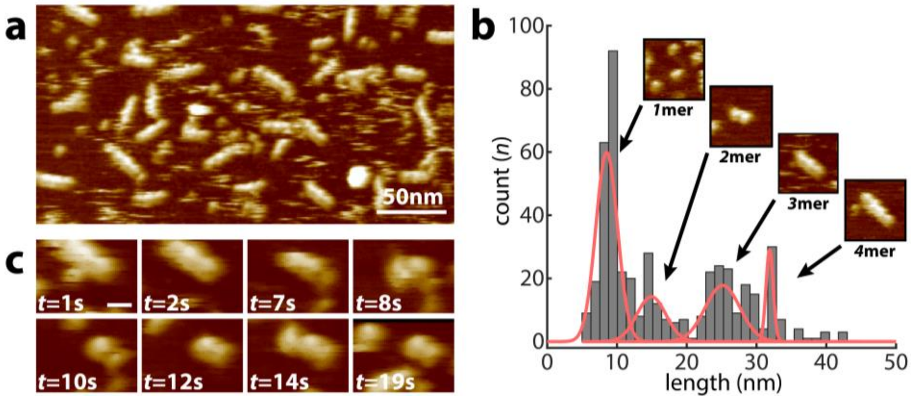

Figure S1) Association of septin monomers into rods in absence of monovalent ions. (**a**) Septin subunit assembly in KCl-free buffer: The assembly of the hetero-octameric rod is characterized by 4 topographic units, each of ~8 nm in length. (**b**) Septin dimers, tetramers, hexamers and octamers with roughly 8, 16, 24, and 32 nm in length, are represented by rods of 1, 2, 3 or 4 topographic features, where topographic monomers, dimers and trimers correspond to 2, 4 and 6 Cdc and the tetramers to 8 Cdc, *i.e.* the septin rod. Note, the septin rod gave the sharpest length distribution, while the monomers, dimers and trimers gave wider distributions and may comprise assemblies of 1 to 3, 3 to 5 and 5 to 7 Cdc, respectively. (**c**) HS-AFM movie frames (time points are indicated in the bottom left corner) showing septin subunit association and dissociation events (scale bar: 10 nm, Supplementary Movies M2, M3).

Supplementary Figure 2)

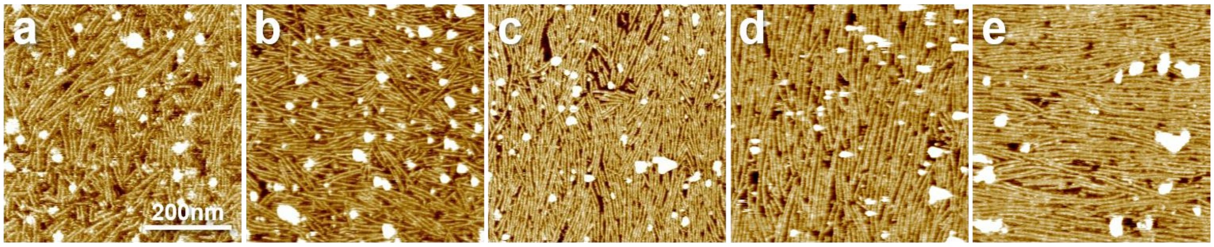

Figure S2) Septin assembly under varying KCl concentrations revealed by HS-AFM. (**a**-**e**) Septin filaments formed and fully covered the surface using 100 nM septin incubated on mica in presence of 50 mM KCl (**a**), 150 mM KCl (**b**), 300 mM KCl (**c**), 450 mM KCl (**d**), 600 mM KCl (**e**).

Supplementary Figure 3)

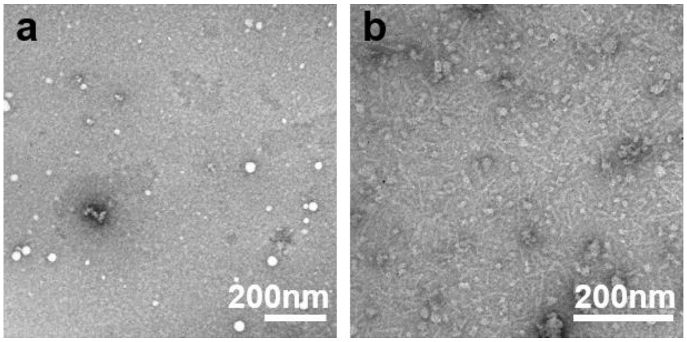

Figure S3) Negative stain EM of septin in varying KCl. (**a**) No septin filaments were found in solution when incubated in high salt buffer. (**b**) Septin filamenst were found from lower salt concentration solution (150 mM KCl).

Supplementary Figure 4)

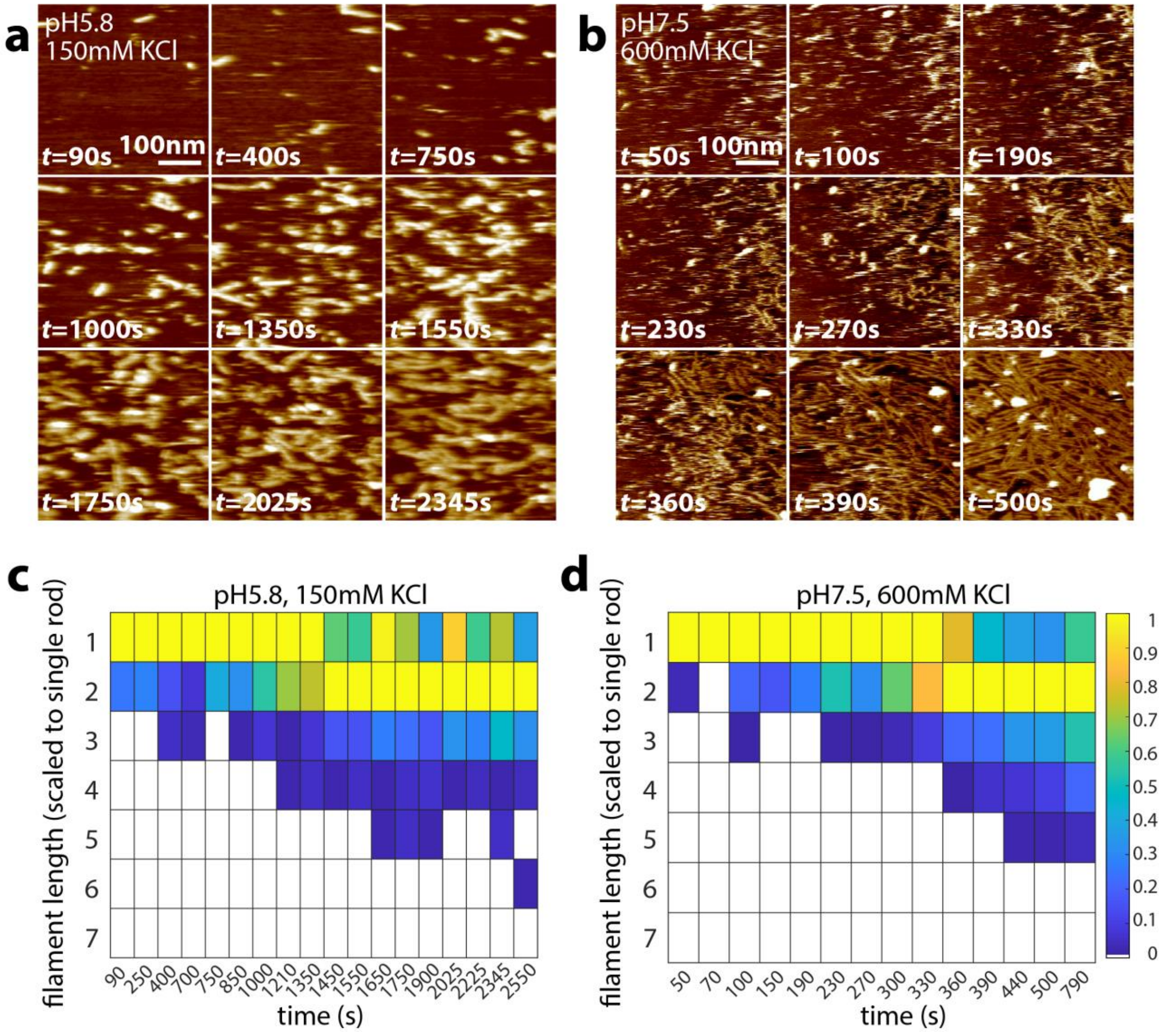

Figure S4) Septin assembly imaged by HS-AFM. (**a, b**) *In situ* HS-AFM frames (top left: imaging conditions; bottom left: time points) of the septin filaments assembly process. (**c, d**) Normalized length histograms of septin assemblies as a function of time. The filament length was scaled to the length of single rod, 32 nm. False color bar: Normalized filament count at each time point.

Supplementary Figure 5)

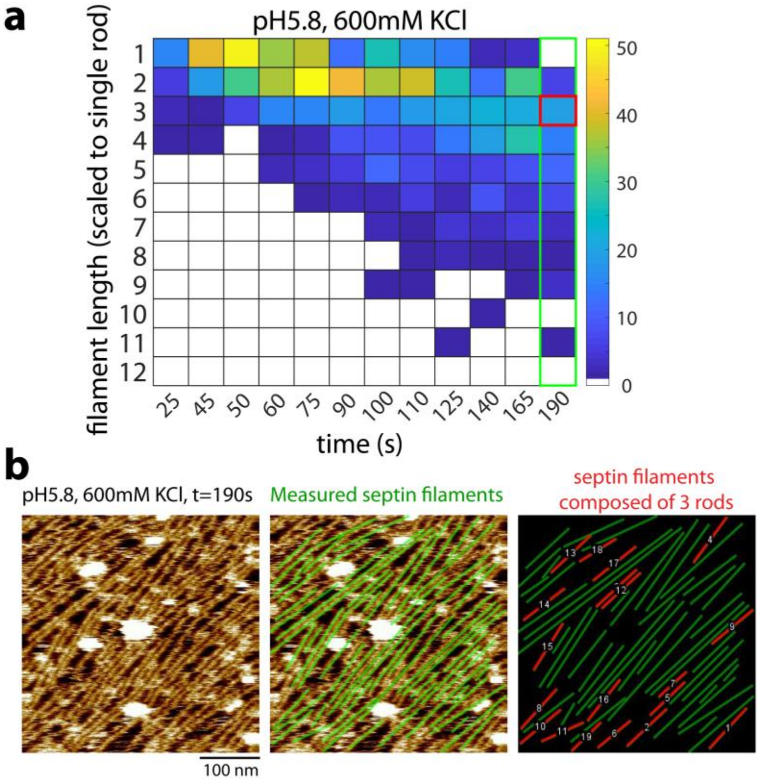

Figure S5) Filament analysis (**a**) Enlarged length histograms of septin assemblies measured at pH 5.8 and 600 mM KCl (see Figure 2 in the main manuscript). (**b**) The *In situ* HS-AFM frame imaged at t=190s (left), the measured septin filaments (middle, green color overlays), and the most populated septin filaments composed of 3 rods highlighted by red color.

Supplementary Figure 6)

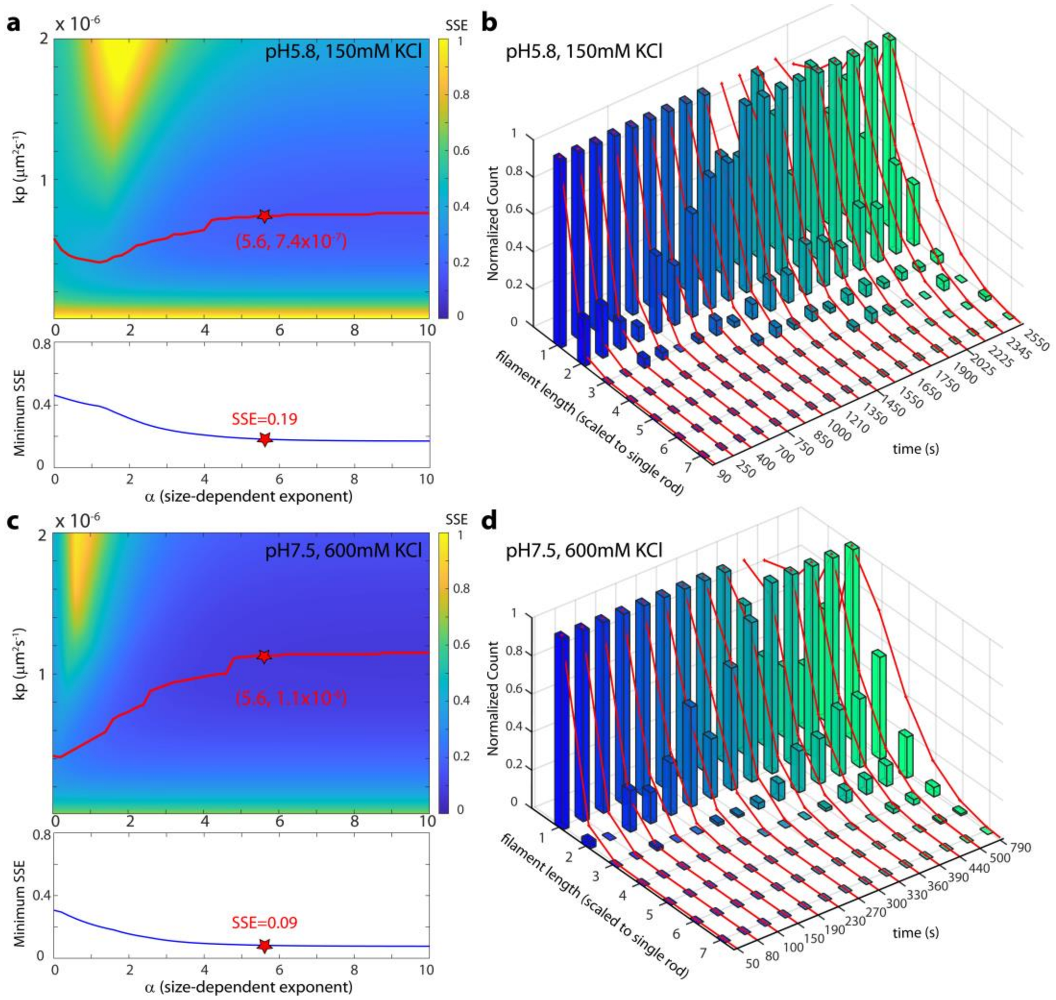

Figure S6) Simulated length histograms based on a diffusion-driven annealing model. (**a, c**) (top) The sum of squared error (SSE) map between experimental and simulated length histograms, which are predicted by using various kp (a diffusion-related constant) and α (a size-dependent factor) values at different conditions (150 mM KCl in pH 5.8 buffer (a), 600 mM KCl in pH 7.5 buffer (b)). The red curve highlights the path of kp at different α values with minimum SSE. (bottom) The minimum SSE at each α value. The red star indicates the best-fit parameters at α=5.6 that offer the global minimum SSE in both conditions. (**b, d**) The comparison between the experimental (color bars) and simulated length histograms (red curves), which are calculated by the best-fit parameters at α=5.6.

Supplementary Figure 7)

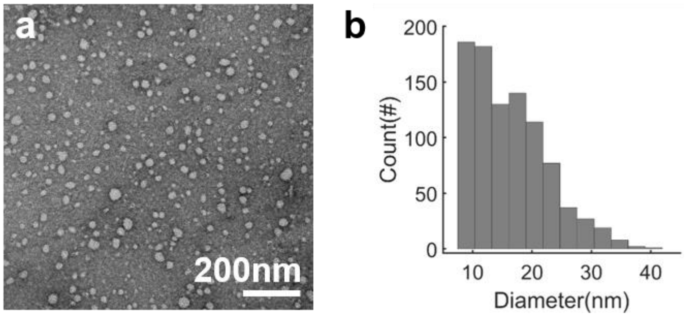

Figure S7) Small lipid clusters co-purified with septins. (**a**) Negative stain EM showed small lipid clusters in the septin sample (see Figure 4 in main text). (**b**) Lipid clusters diameter distribution with average diameter ~15 nm.

Supplementary Figure 8)

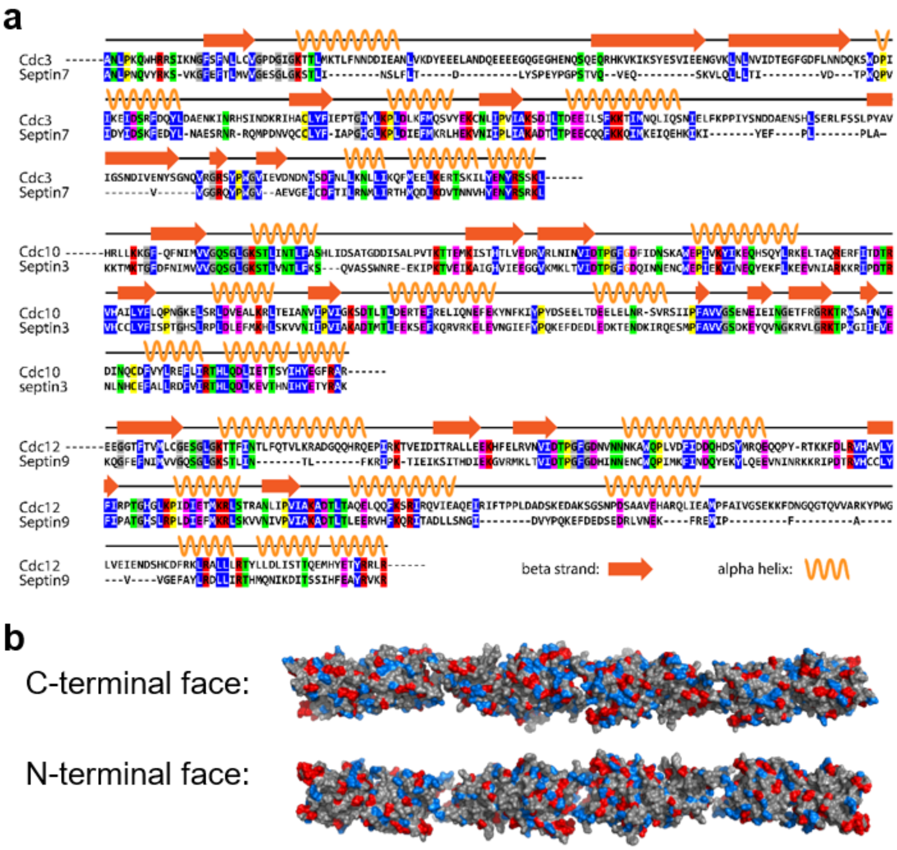

Figure S8) Yeast septin structure prediction and surface charge. (**a**) Cdc3, Cdc10, and Cd12 were predicted and aligned against the known structure of human septin 7 (PDB ID: 2QAG), septin 3 (PDB ID: 4Z54), and septin 9 (PDB ID: 5CYP), all of the alignment proteins show around 50% of sequence identity. (**b**) The yeast septin hetero-octamer structure by aligning of the crystal structure of Cdc11 (PDB ID: 5AR1), and models of Cdc12, Cdc3, and Cdc10, to the crystal structures of human septin 9 (PDB ID: 5CYP), septin 7 (PDB ID: 2QAG), and septin 3 (PDB ID: 4Z54) show nearly neutral surface charges on both the C-terminal and N-terminal faces.

Supplementary Table 1)

Condition	a (%)	a (surface density, μm^{-2})	b	c (s)	R-square
pH5.8, 600mM KCl	90.1±0.4	7038±31	0.027±0.001	107±2	0.998
pH5.8 150mM KCl	82.4±1.0	6438±78	0.002±0.0001	2082±25	0.992
pH7.5, 600mM KCl	84.7±0.9	6619±70	0.010±0.0001	311±7	0.988
pH7.5 150mM KCl	73.6±0.8	5749±63	0.004±0.0001	587±8	0.995

Table S1) Septin surface coverage fitted by a sigmoidal curve ($\frac{a}{1+e^{-b(t-c)}}$, within 95% confidence of bounds) under varying experimental conditions. Given the dimensions of single septin rod (32nm x 4nm, occupied surface area) and the HS-AFM scanning area (400nm x 400nm), we can further estimate the saturated septin surface density (μm^{-2}) from the septin surface coverage (%).

Supplementary Table 2)

Condition	α	k_P (μm^{2}s^{-1})	Minimum SSE
pH5.8, 600mM KCl	5.6	1.8×10^{-5}	0.19
pH5.8 150mM KCl	5.6	7.4×10^{-7}	0.18
pH7.5, 600mM KCl	5.6	1.1×10^{-6}	0.09
pH7.5 150mM KCl	5.6	9.1×10^{-7}	0.32

Table 2) Summary of the best-fit parameters at α=5.6 that offers the global/semi-global minimum SSE in all conditions.

Supplementary Table 3)

Name	Sequence
AGO25149_CDC11REV with Asc1	catgcatttacttataatggcgcgccctattcacctgataatgcctcgg
AGO2515_CDC11 FOR at SnaB1	acgaagattacgaaaccaatatgtacctac

Table 3) Primers used to generate Cdc11 delta CTE septin complex.
