# Supplementary figures and images for: The hierarchical assembly of septins revealed by high-speed AFM
Source: Nat Commun. 2020 Oct 8;11:5062. doi: 10.1038/s41467-020-18778-x (PMC7545167; doi:10.1038/s41467-020-18778-x)

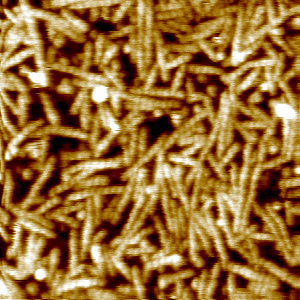

Supplement: Supplementary file 10 — Source Data [file 41467_2020_18778_MOESM10_ESM.zip › Septin source data/Figure 1/Fig. 1a.tif]

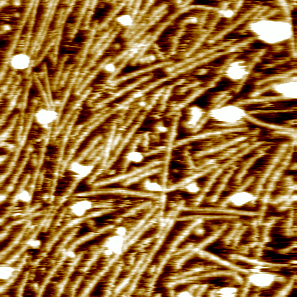

Supplement: Supplementary file 10 — Source Data [file 41467_2020_18778_MOESM10_ESM.zip › Septin source data/Figure 1/Fig. 1b.tif]

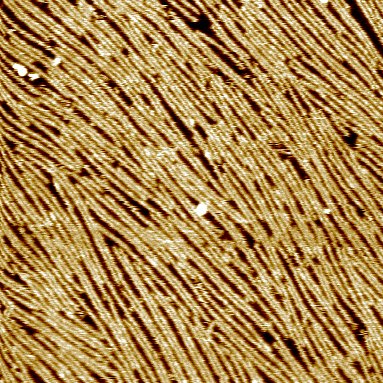

Supplement: Supplementary file 10 — Source Data [file 41467_2020_18778_MOESM10_ESM.zip › Septin source data/Figure 1/Fig. 1c.jpg]

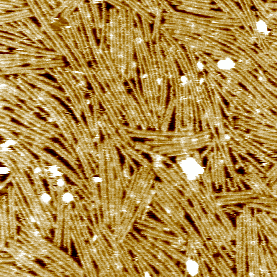

Supplement: Supplementary file 10 — Source Data [file 41467_2020_18778_MOESM10_ESM.zip › Septin source data/Figure 1/Fig. 1d.tif]

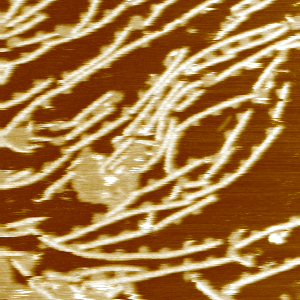

Supplement: Supplementary file 10 — Source Data [file 41467_2020_18778_MOESM10_ESM.zip › Septin source data/Figure 4/Fig. 4a.tif]

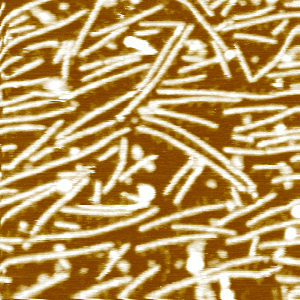

Supplement: Supplementary file 10 — Source Data [file 41467_2020_18778_MOESM10_ESM.zip › Septin source data/Figure 4/Fig. 4b-1.tif]

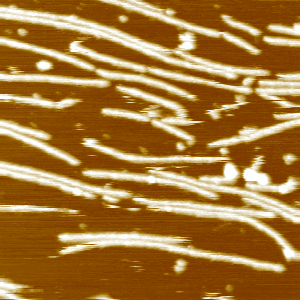

Supplement: Supplementary file 10 — Source Data [file 41467_2020_18778_MOESM10_ESM.zip › Septin source data/Figure 4/Fig. 4b-2.tif]

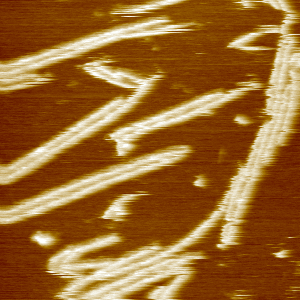

Supplement: Supplementary file 10 — Source Data [file 41467_2020_18778_MOESM10_ESM.zip › Septin source data/Figure 4/Fig. 4b-3.tif]

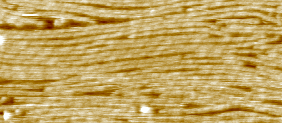

Supplement: Supplementary file 10 — Source Data [file 41467_2020_18778_MOESM10_ESM.zip › Septin source data/Figure 5/Fig. 5a.tif]

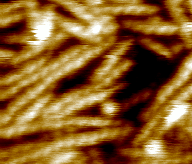

Supplement: Supplementary file 10 — Source Data [file 41467_2020_18778_MOESM10_ESM.zip › Septin source data/Figure 5/Fig. 5c.tif]

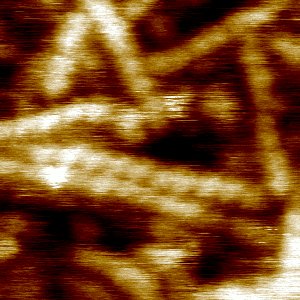

Supplement: Supplementary file 10 — Source Data [file 41467_2020_18778_MOESM10_ESM.zip › Septin source data/Figure 5/Fig. 5d.jpg]

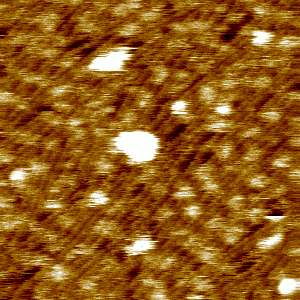

Supplement: Supplementary file 10 — Source Data [file 41467_2020_18778_MOESM10_ESM.zip › Septin source data/Figure 6/Fig. 6a.tif]

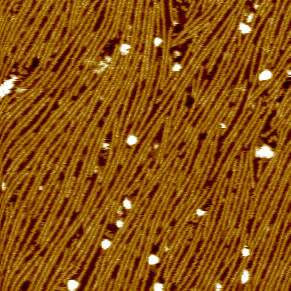

Supplement: Supplementary file 10 — Source Data [file 41467_2020_18778_MOESM10_ESM.zip › Septin source data/Figure 6/Fig. 6c_0min.tif]

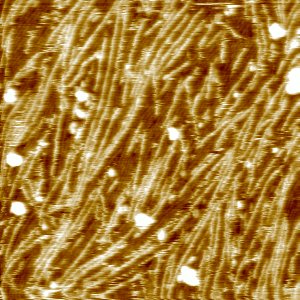

Supplement: Supplementary file 10 — Source Data [file 41467_2020_18778_MOESM10_ESM.zip › Septin source data/Figure 6/Fig. 6c_130min.jpg]

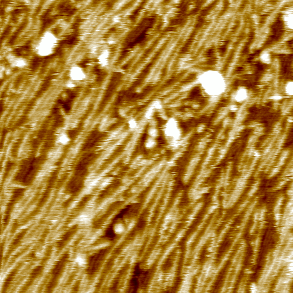

Supplement: Supplementary file 10 — Source Data [file 41467_2020_18778_MOESM10_ESM.zip › Septin source data/Figure 6/Fig. 6c_250min.tif]

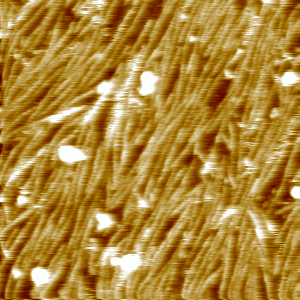

Supplement: Supplementary file 10 — Source Data [file 41467_2020_18778_MOESM10_ESM.zip › Septin source data/Figure 6/Fig. 6c_310min.tif]

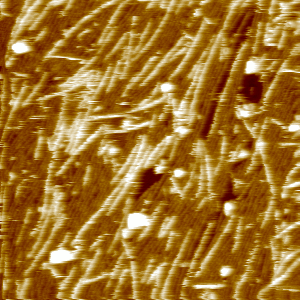

Supplement: Supplementary file 10 — Source Data [file 41467_2020_18778_MOESM10_ESM.zip › Septin source data/Figure 6/Fig. 6c_70min.tif]

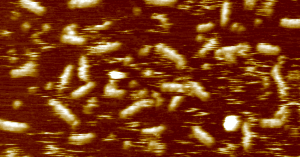

Supplement: Supplementary file 10 — Source Data [file 41467_2020_18778_MOESM10_ESM.zip › Septin source data/Figure SI/Fig. S1/Fig. S1a.tif]

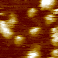

Supplement: Supplementary file 10 — Source Data [file 41467_2020_18778_MOESM10_ESM.zip › Septin source data/Figure SI/Fig. S1/Fig. S1b_1mer.tif]

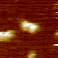

Supplement: Supplementary file 10 — Source Data [file 41467_2020_18778_MOESM10_ESM.zip › Septin source data/Figure SI/Fig. S1/Fig. S1b_2mer.tif]

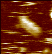

Supplement: Supplementary file 10 — Source Data [file 41467_2020_18778_MOESM10_ESM.zip › Septin source data/Figure SI/Fig. S1/Fig. S1b_3mer.tif]

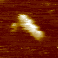

Supplement: Supplementary file 10 — Source Data [file 41467_2020_18778_MOESM10_ESM.zip › Septin source data/Figure SI/Fig. S1/Fig. S1b_4mer.tif]

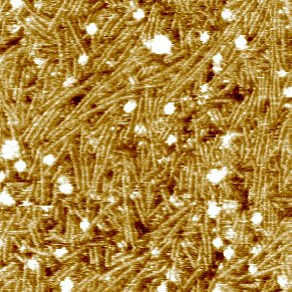

Supplement: Supplementary file 10 — Source Data [file 41467_2020_18778_MOESM10_ESM.zip › Septin source data/Figure SI/Fig. S2/Fig. S2a.tif]

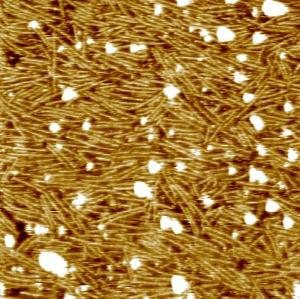

Supplement: Supplementary file 10 — Source Data [file 41467_2020_18778_MOESM10_ESM.zip › Septin source data/Figure SI/Fig. S2/Fig. S2b.tif]

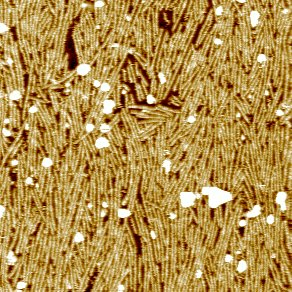

Supplement: Supplementary file 10 — Source Data [file 41467_2020_18778_MOESM10_ESM.zip › Septin source data/Figure SI/Fig. S2/Fig. S2c.tif]

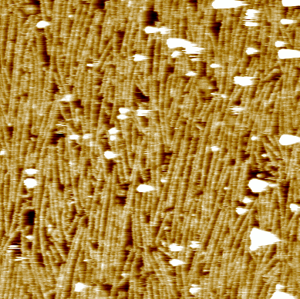

Supplement: Supplementary file 10 — Source Data [file 41467_2020_18778_MOESM10_ESM.zip › Septin source data/Figure SI/Fig. S2/Fig. S2d.tif]

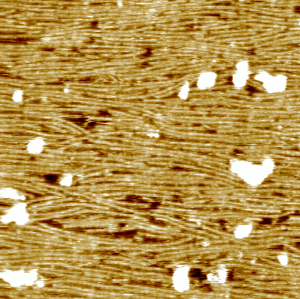

Supplement: Supplementary file 10 — Source Data [file 41467_2020_18778_MOESM10_ESM.zip › Septin source data/Figure SI/Fig. S2/Fig. S2e.tif]

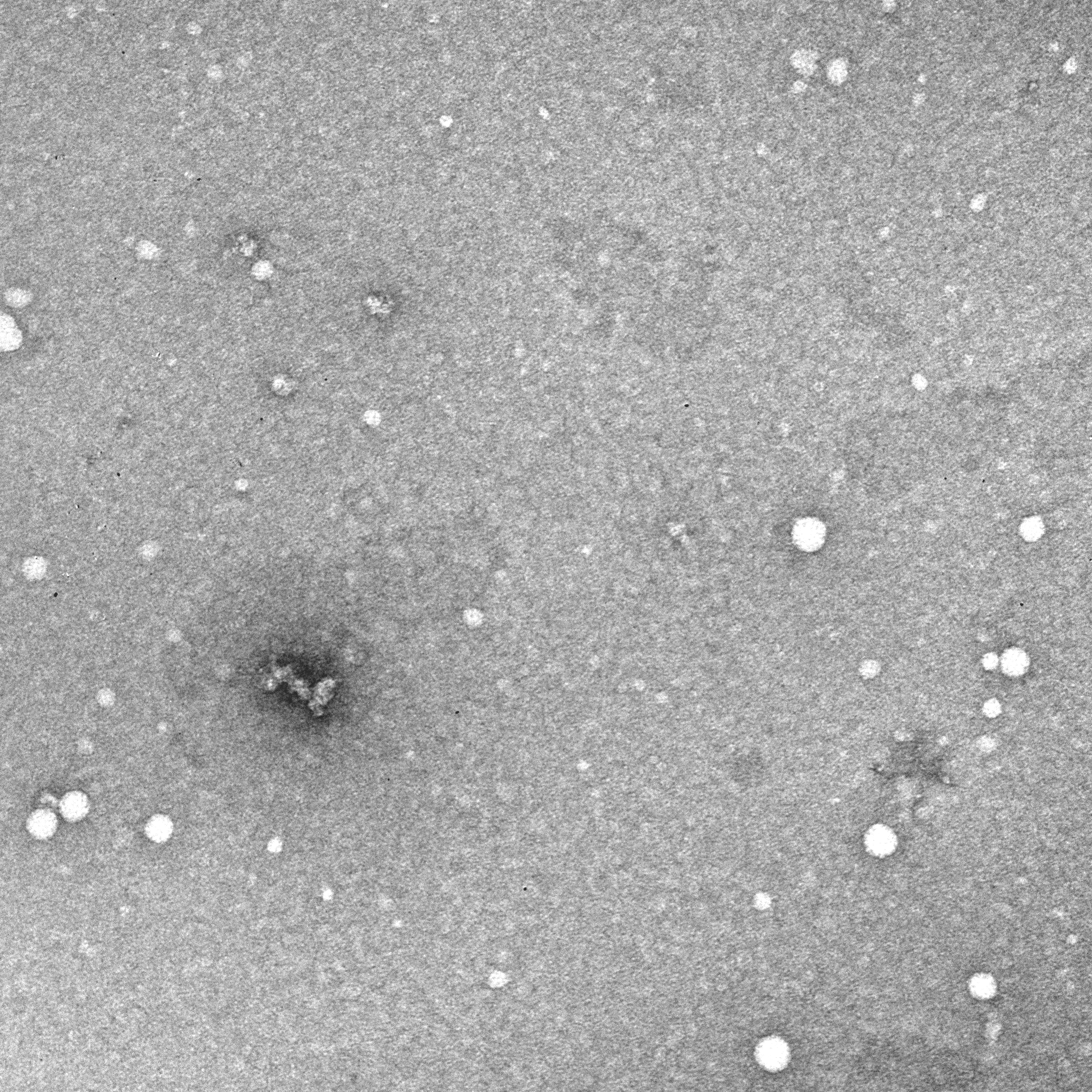

Supplement: Supplementary file 10 — Source Data [file 41467_2020_18778_MOESM10_ESM.zip › Septin source data/Figure SI/Fig. S3/Fig. S3a.tif]

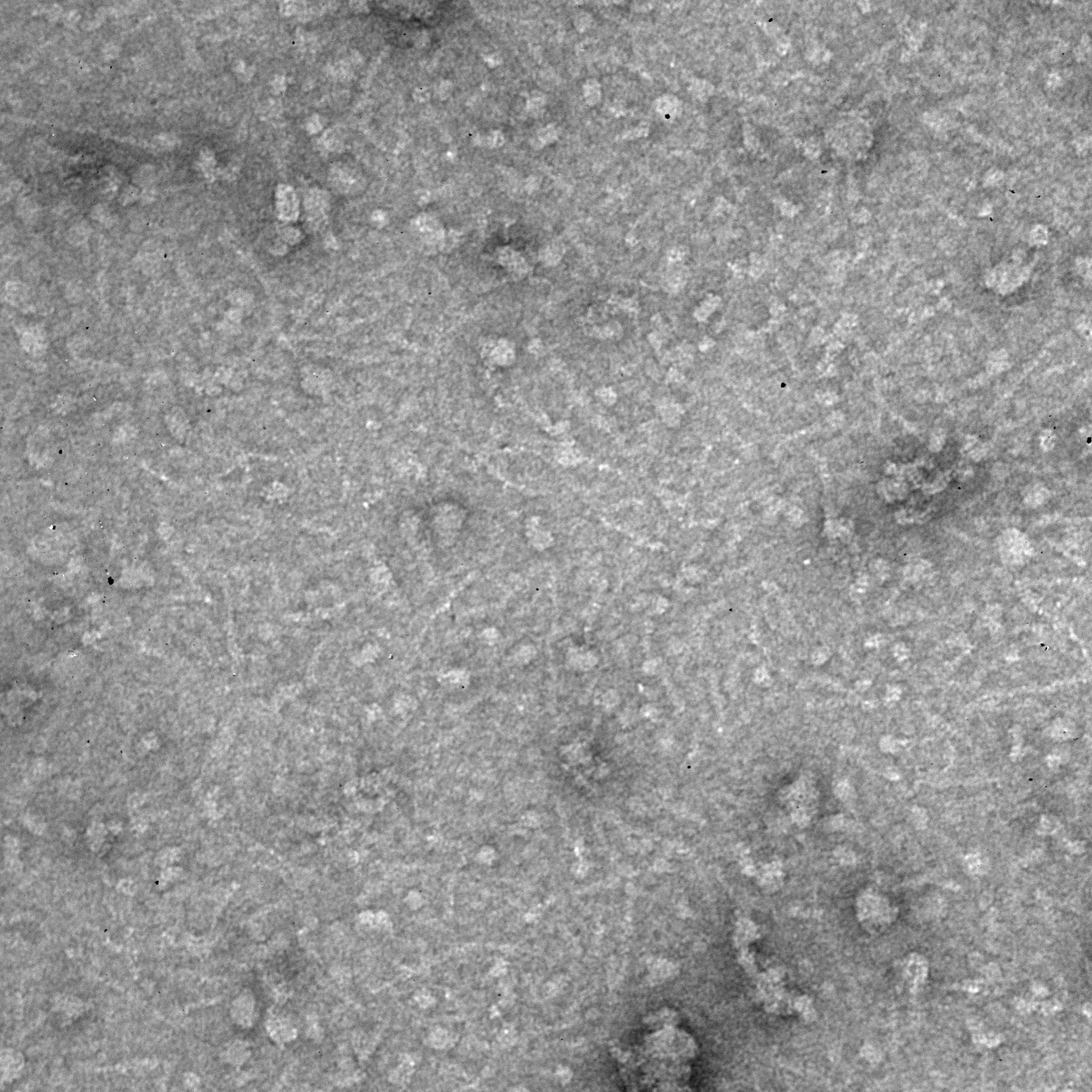

Supplement: Supplementary file 10 — Source Data [file 41467_2020_18778_MOESM10_ESM.zip › Septin source data/Figure SI/Fig. S3/Fig. S3b.tif]

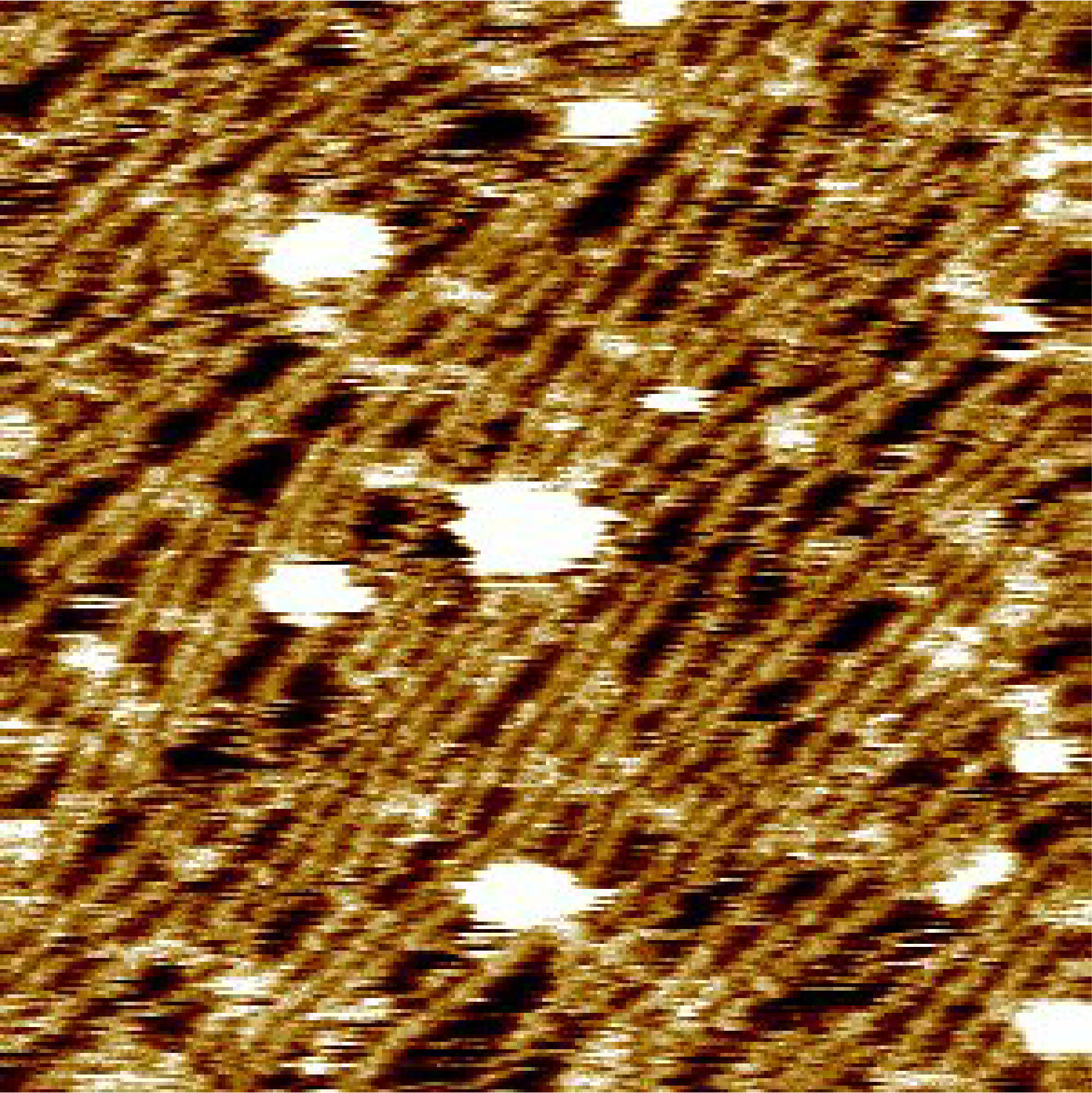

Supplement: Supplementary file 10 — Source Data [file 41467_2020_18778_MOESM10_ESM.zip › Septin source data/Figure SI/Fig. S5/fig. S5.jpg]

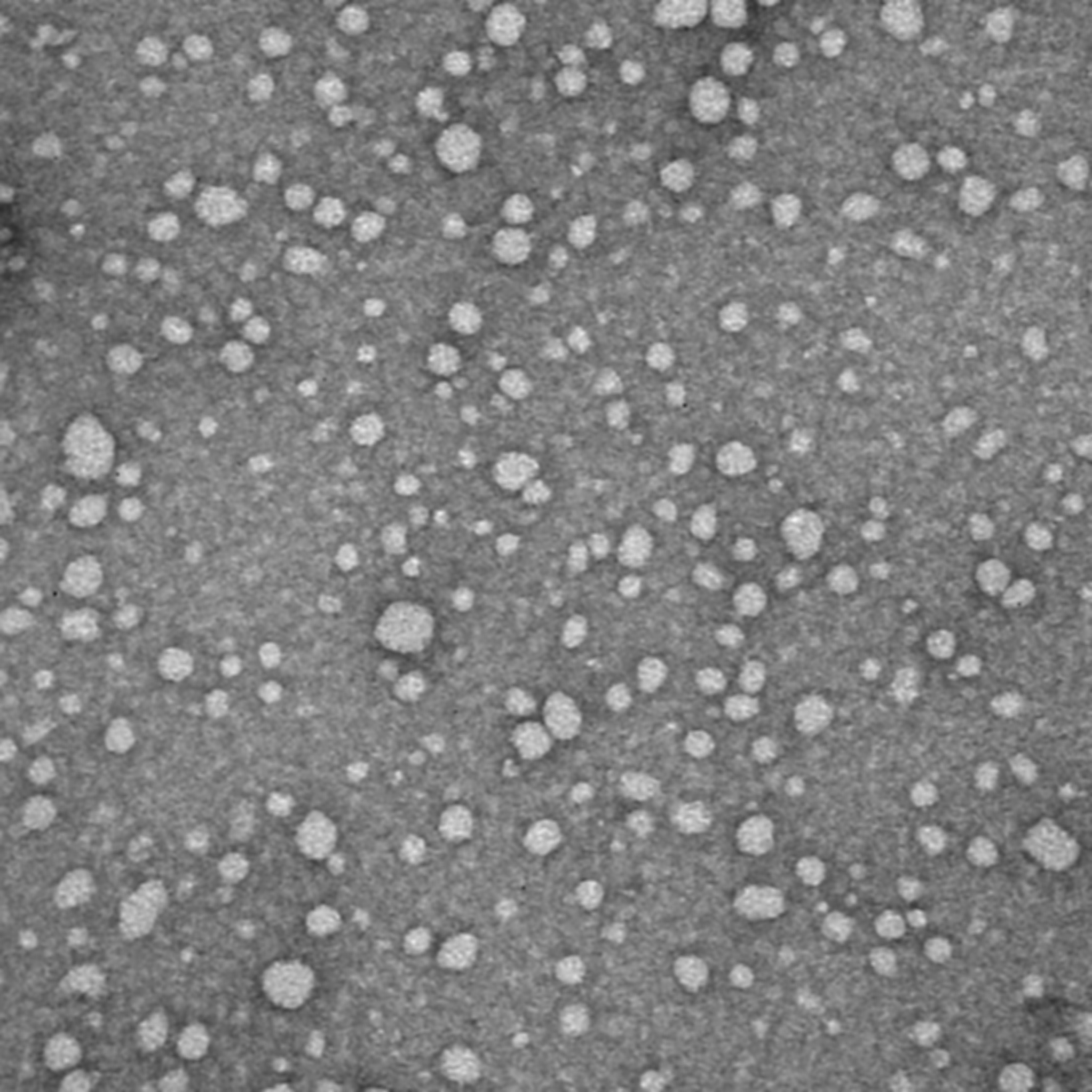

Supplement: Supplementary file 10 — Source Data [file 41467_2020_18778_MOESM10_ESM.zip › Septin source data/Figure SI/Fig. S7/Fig. S7a.tif]

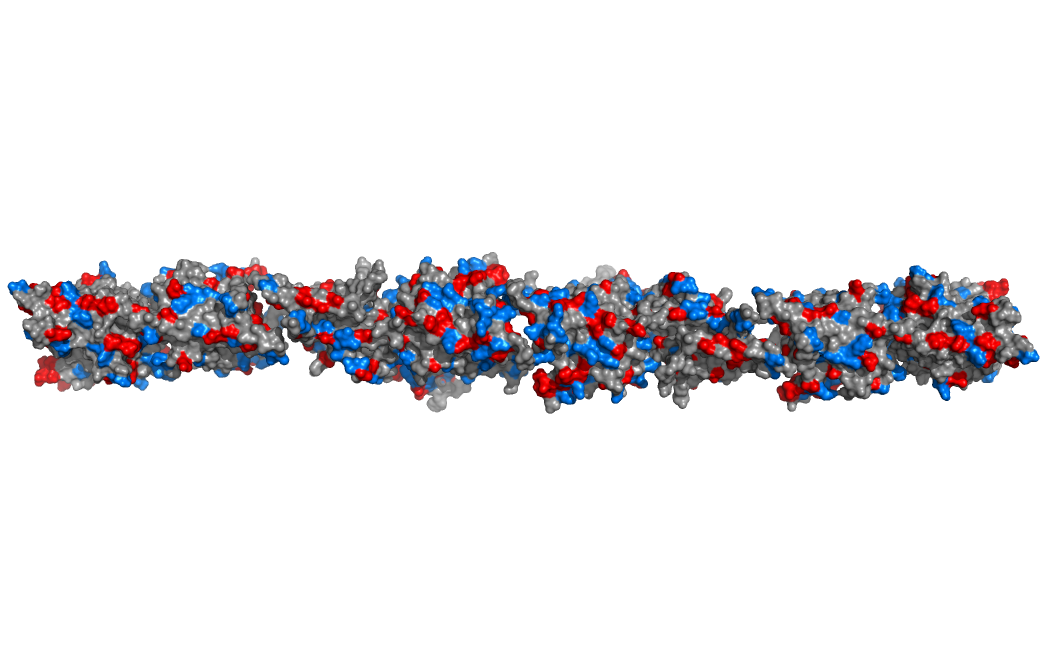

Supplement: Supplementary file 10 — Source Data [file 41467_2020_18778_MOESM10_ESM.zip › Septin source data/Figure SI/Fig. S8/C-terminal face.png]

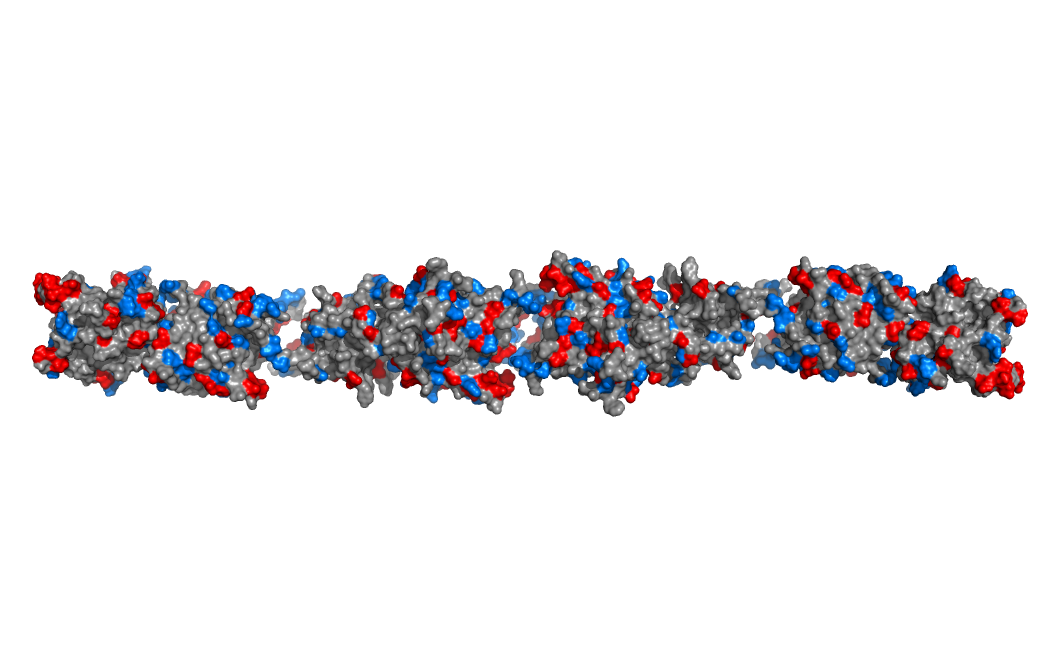

Supplement: Supplementary file 10 — Source Data [file 41467_2020_18778_MOESM10_ESM.zip › Septin source data/Figure SI/Fig. S8/N-termianl face.png]
